# Some extensions in continuous models for immunological correlates of protection

**DOI:** 10.1186/s12874-015-0096-9

**Published:** 2015-12-28

**Authors:** Andrew J. Dunning, Jennifer Kensler, Laurent Coudeville, Fabrice Bailleux

**Affiliations:** Sanofi Pasteur, Swiftwater, PA 18370 USA; Virginia Tech, Blacksburg, VA USA; Present address: Shell Global Solutions, Houston, TX 77082 USA; Sanofi Pasteur, Lyon Cedex, 69367 France; Sanofi Pasteur, Marcy l’Étoile, 69280 France

**Keywords:** Vaccine, Correlate of protection, Correlate of immunity, Surrogate endpoint, Immunological assay

## Abstract

**Background:**

A scaled logit model has previously been proposed to quantify the relationship between an immunological assay and protection from disease, and has been applied in a number of settings. The probability of disease was modelled as a function of the probability of exposure, which was assumed to be fixed, and of protection, which was assumed to increase smoothly with the value of the assay.

**Methods:**

Some extensions are here investigated. Alternative functions to represent the protection curve are explored, applications to case-cohort designs are evaluated, and approaches to variance estimation compared. The steepness of the protection curve must sometimes be bounded to achieve convergence and methods for doing so are outlined. Criteria for evaluating the fit of models are proposed and approaches to assessing the utility of results suggested. Models are evaluated by application to sixteen datasets from vaccine clinical trials.

**Results:**

Alternative protection curve functions improved model evaluation criteria for every dataset. Standard errors based on the observed information were found to be unreliable; bootstrap estimates of precision were to be preferred. In most instances, case-cohort designs resulted in little loss of precision. Some results achieved suggested measures for utility.

**Conclusions:**

The original scaled logit model can be improved upon. Evaluation criteria permit well-fitting models and useful results to be identified. The proposed methods provide a comprehensive set of tools for quantifying the relationship between immunological assays and protection from disease.

**Electronic supplementary material:**

The online version of this article (doi:10.1186/s12874-015-0096-9) contains supplementary material, which is available to authorized users.

## Background

Immunological assays measure characteristics of the immune system, such as antibody concentrations or the ability of serum to neutralize pathogens in vitro, which are induced by an immune stimulus such as disease or vaccination, and which are associated with protection from disease. The relationship between an immunological assay and protection from disease is of considerable interest in vaccines research. In early phase clinical trials of a new vaccine, an immune response observed by an immunological assay suggests the possibility the vaccine might be protective. In later phase trials, values of immunological assays are used for dose selection and dose ranging, and to assess the effect of co-administration with other vaccines. In vaccine efficacy trials, data on post-vaccination assay values and subsequent disease occurrence may be used to predict protection in other settings. ‘Correlates of protection’ – threshold values of specific immunological assays believed to be associated with protection from disease – have been established for many vaccine-preventable diseases [1], and are used as surrogates for protection in the development of combination vaccines.

A number of elements need to be demonstrated for an assay to reliably substitute for observation of clinical disease. First it should be shown that increasing assay values correlate with reduction in the rate of disease; standard statistical methods are available for this purpose, such as logistic regression [2], and the assay is then described as a ‘correlate of risk’ [3, 4]. When vaccination both increases assay values and reduces the rate of disease the term ‘correlate of protection’ has been suggested [5]; elsewhere the term ‘correlate of vaccine-induced protection’ has been used when this condition is met [6, 7]. It is desirable to show that an assay meets criteria for a surrogate endpoint, such as the Prentice criteria [8] or that it explains a high proportion of treatment effect [9]. Alternatively or additionally it can be useful to demonstrate that the property measured by the assay is causally or mechanistically related to protection from disease (rather than both merely reflecting some common, unobserved characteristic such as robustness of the immune system). Immunologists understanding of the mechanisms of action of the immune system have shown such mechanistic associations [10–12], and statistical methods have been developed seeking to demonstrate causal associations [13–16]. Finally, it is necessary to quantify the relationship between assay value and protection – the level of protection at each assay value – which is the subject of this research.

Established correlates of protection have been characterized as threshold values of immunological assays; interpretation of such thresholds can however be problematic – whether the threshold is one at which protection can be regarded as complete, or whether it represents a ‘population average’ measure differentiating susceptible from protected individuals – and statistical methods with different interpretations have been developed [17–21]. In reality the relationship is likely continuous. Natural variability between individuals means that at any given assay value some individuals will be protected and some not, and if protection does in fact increase with increasing assay value then the proportion of individuals protected will increase in a smooth continuous manner, which may be represented by a protection curve.

**Fig. 1:**
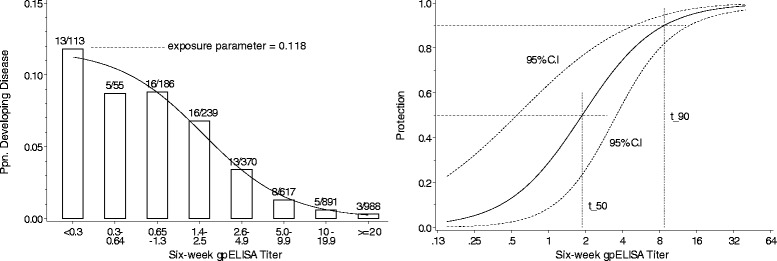
Scaled logit model fitted to the White/varicella data [23]. Left panel: Bars show the number and proportion of subjects who developed disease in each interval of assay value; curve is estimated probability of disease. Right panel: Estimated protection curve with 95 % confidence interval for protection and estimates of assay values at which protection is 50 % and 90 %

Most data on the relationship between assay value and subsequent protection from disease comes from settings in which the exposure of subjects to the pathogen of interest is not guaranteed, so that although standard statistical methods such as logistic regression can demonstrate a relationship between assay values and disease they cannot explicitly quantify protection. Absence of disease may indicate protection, or merely lack of exposure. A scaled logit model modelling disease occurrence as a function of assay value by an exposure parameter and a parametric protection curve has been proposed, using the logistic function of log-assay value to model the protection curve [22]. An illustration of the model fitted to the White/varicella data [23] is shown in Fig. 1.

The model has been used to investigate the association between cell-mediated immunity and protection from influenza [24], between hemagglutination inhibition assay values and protection from influenza [25, 26], and the probability of influenza-like illness in HIV-positive subjects [27], and has been suggested for other applications [28].

The logistic function is however only one of a number of 2-parameter symmetrical sigmoid functions which may be used to represent the protection curve; other functions are here investigated. A generalized symmetrical sigmoid function is developed. Some results suggest the assumption that protection approaches 100 % at high assay values may not hold; models for incomplete protection are considered. Symmetrical sigmoid functions constrain the two halves of the protection curve to be inverted mirror images of each other; asymmetrical sigmoid functions are explored.

In the paper introducing the scaled logit model, convergence failed for two illustrative datasets, and it was hypothesized this was because these assays were not useful as correlates of protection. This has since been found to be a misinterpretation, the lack of convergence being in fact due to the steepness of the protection curve increasing in the likelihood maximization algorithm until it exceeded computer limits. Methods for limiting the steepness of the protection curve and other fitting issues are discussed.

Case-cohort designs have been proposed as an economical approach to assessing correlates of protection when the proportion of subjects developing disease is small [29, 30]. Application of proposed methods to case-cohort designs is explored.

Methods for estimating precision and confidence intervals are examined, and evaluation criteria proposed. Methods are illustrated by application to sixteen datasets from clinical trials.

Much data on the relationship between assay value and disease comes from vaccine efficacy trials. A limitation of such data is that the trial will be powered for the efficacy endpoint and will likely be underpowered for a correlate of protection investigation. Data from such a trial can thus at best support exploratory analysis and yield conclusions of the form: ‘the model found to be the best fit to the data in this case was…’ rather than being confirmatory.

## Methods

In outline, our research:

i) develops a general form of model for the probability of disease as a function of exposure and protection;
ii) identifies and develops alternative functions to represent the protection curve;
iii) proposes criteria for model evaluation and selection;
iv) fits models to sixteen illustrative datasets, calculates evaluation and model selection criteria, and compares methods for estimating standard errors;
v) proposes alternative data analysis strategies.

Data consist of assay values and subsequent disease occurrence in subjects from some defined population; descriptions of the illustrative datasets are provided at the end of this section.

### General form of model incorporating both exposure and protection

If both exposure and susceptibility (absence of protection) are necessary for the development of disease, and together they are sufficient, the probability of disease may be expressed as

$$ \mathrm{P}\left(\mathrm{disease}\right)=\lambda \times \left[1-\pi \left(t;\alpha, \beta, \dots \right)\right] $$

where *λ* represents exposure (or more generally the effect of factors independent of assay value), *π*(⋅) is a smooth increasing sigmoid function into (0,1) representing protection (or more generally the effect of factors associated with assay value) – a ‘protection curve’, *t* is log-assay value and *α* and *β* are location and slope parameters of the protection curve. Other parameters may govern shape, incomplete protection, symmetry, etc. Two parameterizations are possible, *π*(*t*; *α*, *β*, …) = *π*(*α* + *β t*, …) as used in the original scaled logit model or *π*(*t*; *α*, *β*, …) = *π*(*β*(*t* − *α*), …) as introduced by Coudeville [25]. The latter is used here since exp (*α*) is then the assay value at which protection is 50 % in models with symmetrical protection curves. Models are fitted by maximum likelihood.

### Symmetrical two-parameter protection curves

Six 2-parameter symmetrical sigmoid functions which might represent protection curves were explored:

i) the error function, *π*(*β* (*t* − *α*)) = Φ(*β* (*t* − *α*)), where Φ(·) is the cumulative standard normal distribution function;
ii) the logistic function (as in the scaled logit model), $\pi \left(\beta \kern0.1em \left(t-\alpha \right)\right)=\frac{ \exp \left(\beta \kern0.1em \left(t-\alpha \right)\right)}{1+ \exp \left(\beta \kern0.1em \left(t-\alpha \right)\right)}$;
iii) $\pi \left(\beta \kern0.1em \left(t-\alpha \right)\right)=\frac{\beta \kern0.1em \left(t-\alpha \right)}{\sqrt{1+{\left(\beta \kern0.1em \left(t-\alpha \right)\right)}^2}}\div 2+{\scriptscriptstyle \frac{1}{2}}$, this is referred to as the square root sigmoid function;
iv) the double exponential function, $$ \pi \left(\beta \kern0.1em \left(t-\alpha \right)\right)=1\left(\beta \kern0.1em \left(t-\alpha \right)\ge 0\right)\times \left[1-{\scriptscriptstyle \frac{1}{2}} \exp \left(-\beta \kern0.1em \left(t-\alpha \right)\right)\right] $$$$ +1\left(\beta \kern0.1em \left(t-\alpha \right)<0\right)\times \left[{\scriptscriptstyle \frac{1}{2}} \exp\;\left(\beta \kern0.1em \left(t-\alpha \right)\right)\right], $$ where 1(⋅) is the indicator function taking the value 1 when its argument is true and 0 when it is false;
v) the arctangent function, $\pi \left(\beta \kern0.1em \left(t-\alpha \right)\right)={\scriptscriptstyle \frac{1}{\pi }}\mathrm{t}\mathrm{a}{\mathrm{n}}^{-1}\left(\beta \kern0.1em \left(t-\alpha \right)\right)+{\scriptscriptstyle \frac{1}{2}}$;
vi) $\pi \left(\beta \kern0.1em \left(t-\alpha \right)\right)=\frac{\beta \kern0.1em \left(t-\alpha \right)}{1+\left|\beta \kern0.1em \left(t-\alpha \right)\right|}\div 2+{\scriptscriptstyle \frac{1}{2}}$, this is referred to as the absolute sigmoid function.

The principal difference between the functions is that for a given slope at the inflection point the error function approaches the asymptotes most quickly, with the logistic, square root, arctangent and absolute sigmoid functions approaching progressively more slowly; the double exponential function approaches more slowly near the inflection point but more quickly in the tails. By way of illustration, if the arctangent function was used for the protection curve the model to be fitted would be

$$ \mathrm{P}\left(\mathrm{disease}\right)=\lambda \times \left[1-\left({\scriptscriptstyle \frac{1}{\pi }}\mathrm{t}\mathrm{a}{\mathrm{n}}^{-1}\left(\beta \kern0.1em \left(t-\alpha \right)\right)+{\scriptscriptstyle \frac{1}{2}}\right)\right] $$

Models with each of the six functions were fitted to the sixteen illustrative datasets.

### A generalized symmetrical protection curve

Consideration of the algebraic expressions for the absolute sigmoid and square root sigmoid functions suggests a generalized symmetrical sigmoid function

$$ \pi \left(\beta \left(t-\alpha \right)\right)=\frac{\beta \left(t-\alpha \right)}{{\left(1+{\left|\beta \left(t-\alpha \right)\right|}^{\kappa}\right)}^{1/\kappa }}\div 2+{\displaystyle \frac{1}{2}} $$

where *κ* is a shape parameter governing how fast the curve approaches the asymptotes for a given slope at the inflection point. When *κ*=1 the function is the absolute sigmoid function and when *κ*=2 the function is the square root sigmoid function; when *κ*=1.5 the function approximates the arctangent function, when *κ*=2.9 it approximates the logistic function, and when *κ*=3.4 it approximates the error function. *κ* is estimated in the likelihood maximization. Models with generalized symmetrical protection curves were fitted to each of the illustrative datasets.

### ‘Incomplete protection’ protection curves

Inspection of plots of estimated generalized symmetric protection curves indicated that for some datasets protection did not approach 100 % even at the highest assay values, prompting investigation of a model where the maximum protection was less than 1.

$$ \mathrm{P}\left(\mathrm{disease}\right)=\lambda \times \left[1-\gamma \pi \left(\beta \left(t-\alpha \right)\right)\right] $$

where *γ* ∈(0,1] is the maximum level of protection and *π*(⋅) is one of the six 2-parameter symmetrical sigmoid functions. *γ* is estimated in the likelihood maximization. Models were fitted to each of the sixteen illustrative datasets.

### Non-symmetrical protection curves

Two approaches to relaxing the symmetricality of the protection curve were explored. An alternative to setting *t* = log(*t*_N_) where *t*_N_ is the assay value on the natural scale is the transformation *t*_*ν*_ = *t*_N_^*ν*^ log(*t*_N_). Using *t*_*ν*_ with any of the 2-parameter symmetrical functions has the effect of making the protection curve asymmetrical relative to *t* = log(*t*_N_). For example, if the error function was used the model to be fitted would be

$$ \mathrm{P}\left(\mathrm{disease}\right)=\lambda \times \left[1-\Phi \left(\beta \kern0.1em \left({t}_N^{\nu } \log \left({t}_N\right)-\alpha \right)\right)\right] $$

To ensure the transformation remains strictly increasing requires that *ν* sometimes be bounded: if *t*_N,min_ < 1 then *ν* < −1/log(*t*_N,min_), and if *t*_N,max_ > 1 then *ν* > −1/log(*t*_N,max_). Models using the transformation with error and absolute sigmoid protection functions (the 2-parameter functions approaching the asymptotes most quickly and most slowly) were fitted to the illustrative datasets.

A notionally straightforward approach to more flexibly modeling the lower left and upper right parts of the protection curve is a two-part splined model [31] consisting of two curves, each with their own exposure, location and slope parameters, but constrained to join smoothly at a ‘knot’.

$$ \mathrm{P}\left(\mathrm{disease}\right)=1\left(t<{t}_{knot}\right)\;{\lambda}_1\left[1-\pi \left({\beta}_1\left(t-{\alpha}_1\right)\right)\right]+1\left(t>{t}_{knot}\right){\lambda}_2\left[1-\pi \left({\beta}_2\left(t-{\alpha}_2\right)\right)\right] $$

subject to the constraints

$$ \begin{array}{c}{\lambda}_1\left[1-\pi \left({\beta}_1\left({t}_{knot}-{\alpha}_1\right)\right)\right]={\lambda}_2\left[1-\pi \left({\beta}_2\left({t}_{knot}-{\alpha}_2\right)\right)\right]\\ {}{\left.\frac{d}{dt}{\lambda}_1\left[1-\pi \left({\beta}_1\left(t-{\alpha}_1\right)\right)\right]\;\right|}_{t={t}_{knot}}={\left.\frac{d}{dt}{\lambda}_2\left[1-\pi \left({\beta}_2\left(t-{\alpha}_2\right)\right)\right]\;\right|}_{t={t}_{knot}}\end{array} $$

where subscripts ⋅_1_ and ⋅_2_ refer to the lower left and upper right parts of the protection curve respectively. Since the likelihood is not continuous in *t*_*knot*_ a profile likelihood is constructed over candidate values of *t*_*knot*_ and the maximum chosen. Two-part spline models were developed with error function and absolute sigmoid protection curves; details are given in Additional file 1 at the publisher’s web site. Models were fitted to the sixteen datasets.

### Case-cohort designs

In case-cohort designs, the likelihood is created by fitting

$$ \mathrm{P}\left(\mathrm{disease}\Big|t\ \mathrm{measured}\right)=\frac{1}{1+\rho \kern0.28em \left({\displaystyle \frac{1}{\lambda \left[1-\pi \left(t;\alpha, \beta, \dots \right)\right]}-1}\right)} $$

to the data, where *ρ* is the non-case fraction, i.e. the proportion of non-cases whose assay values are measured. A question of interest is whether the precision of parameter estimates is lessened by the reduced information for non-cases. The question was investigated for ten illustrative datasets in which the number of cases was small relative to the total number of subjects. From each illustrative dataset, 300 case-cohort datasets comprising the cases and a random sample of the non-cases, numbering 20×, 10× and 5× the number of cases, 100 of each, were generated, and case-cohort models with error function and absolute sigmoid protection curves (the curves approaching the asymptotes most quickly and most slowly) fitted. Interest centers on the precision of the estimate of the location parameter *α*, since the assay value at which protection is 50 %, 80 % or other percentage is a function of *α*. The standard error of the location parameter $\widehat{\alpha}$ estimated in the case-cohort datasets from the observed information was compared to its standard error in the ‘all subjects’ illustrative datasets.

### Standard errors and confidence intervals

Standard errors based on the observed information were evaluated and compared with bootstrap estimates [32]. Four estimation methods were compared in two models:

i) the median of the standard errors based on the observed information returned by fitting the model to 1000 non-parametric bootstrap datasets;
ii) the standard deviation of the parameter estimates found by fitting the model to the 1000 non-parametric bootstrap datasets;
iii) the median of the standard errors based on the observed information returned by fitting the model to 1000 parametric bootstrap datasets;
iv) the standard deviation of the parameter estimates found by fitting the model to the 1000 parametric bootstrap datasets.

Methods were evaluated by fitting models with error function and absolute sigmoid protection curves to bootstrap datasets from the 27 combinations of protection curve and illustrative dataset for which maximum likelihood estimates (MLEs) had been found. Parametric bootstrap datasets were generated using the parameter estimates from the illustrative datasets as parameter values.

Again, interest centers on the precision of the estimate of the location parameter *α*, or more particularly on the precision of a measure such as the assay value at which protection is 50 %; the utility of the estimate of such a measure might be assessed by the percentage of subjects with assay values within and outside the 95 % confidence interval for the measure.

The variability in standard errors based on the observed information was examined, estimates by the four methods were compared, the coverage probability of 95 % two-sided confidence intervals calculated from the observed information in the parametric bootstrap datasets was assessed, and the percentages of subjects falling within and outside 95 % confidence intervals for the assay value at which protection is 50 % were calculated.

### Limiting the steepness of the protection curve, maximum likelihood estimates, and evaluation criteria

The likelihood maximization algorithm can fail to converge if the steepness of the protection curve increases until the slope parameter exceeds computer limits, i.e. if the protection curve approaches a step function. This may be controlled for by bounding the slope parameter in the maximization algorithm. A reasonable bound might be one where protection increased from 1 to 99 % in some small fraction of the range of assay values, say 1/50^th^; the curve would then be virtually indistinguishable from a step function. Such a bound would be

$$ \beta \le 2{\uppi}^{-1}(0.99)\times 50\div \mathrm{range}\left( \log \hbox{-} \mathrm{assay}\ \mathrm{value}\right) $$

for symmetrical curves or *β* ≤ [*π*^− 1^(0.99) − *π*^− 1^(0.01)] × 50 ÷ range(log‐assay value) more generally, where *π*^−1^(⋅) is the inverse of the protection function. There would seem to be no reason not to routinely bound the slope when fitting models.

For each model fitting, MLEs were considered to have been found if the fitting algorithm converged, the Hessian matrix was positive definite, and $0<\widehat{\lambda}<1$. It was noted that different starting values for parameters could lead to convergence at different points so a variety of starting values were used. The convergence criteria of the fitting algorithm used was tightened to ensure convergence to the same point from different starting values, and the Hessian matrix was considered positive definite if all eigenvalues were greater than −10^−4^. Details are given in Additional file 2.

The primary criterion for evaluating alternative models was −2×log-likelihood. Goodness-of-fit was calculated by the method of Hosmer and Lemeshow [2, 33]. Since the estimated protection curve is conditional on *λ*, models with low coefficients of variation of $\widehat{\lambda}$ may be preferred, and this was calculated for each fitted model based on the observed information.

### Illustrative datasets

The various methods were evaluated by application to 16 datasets from five clinical trials:

- German pertussis datasets: eight assays and occurrence of disease from a sub-study of a pertussis vaccine efficacy trial conducted in Germany between 1991 and 1994 [34];
- Piedra/RSV datasets: assays for antibody to RSV/A and RSV/B among subjects presenting with acute respiratory symptoms at a hospital in Texas, and subsequent disease confirmation [35];
- White/varicella dataset: varicella glycoprotein assay for children vaccinated with varicella vaccine in clinical trials conducted between 1987 and 1989, and disease occurrence in the following 12 months (reconstructed from published data) [23];
- Swedish pertussis datasets: four assays from subjects potentially exposed to pertussis by another household member and their subsequent development of disease, from a sub-study of a vaccine efficacy trial conducted in Sweden between 1992 and 1995 [36];
- Black Nicolay HAI dataset: post-vaccination hemagglutination inhibition assay (HAI) titers to H3N2 influenza and the occurrence of H3N2 influenza among children 6 to 72 months of age in the following influenza season (reconstructed from published data) [26].

Code to create the datasets reconstructed from published sources, and to fit the symmetrical two-parameter protection curve, generalized symmetrical protection curve, ‘incomplete protection’ protection curve and *t*_N_^*ν*^ log(*t*_N_) transformation non-symmetrical protection curve models is provided in Additional file 3.

## Results

Results are intended to illustrate the statistical methods proposed and are not intended to be interpreted as scientifically valid immunological conclusions.

### Symmetrical protection curves

The evaluation criteria for the six models with 2-parameter symmetrical protection curves when fitted to the sixteen illustrative datasets are shown in Table 1, together with the rank of the model by each evaluation criterion.

**Table 1:** Evaluation criteria for models for which MLEs were found with 2-parameter symmetrical protection curves fitted to illustrative datasets

−2×log-likelihood (rank)						
Goodness-of-fit (rank)						
Coef. of Var. $\widehat{\lambda}$ (rank)	Protection curve/function					
Dataset (cases of disease:subjects)	Error function	Logistic function	Square root sigmoid	Double exponential	Arctangent function	Absolute sigmoid function
German pertussis FHA IgG (44:1988)	375.441 (6) 0.1799 (1) 0.4365 (6)	375.247 (5) 0.1645 (3) 0.3247 (5)	374.949 (4) 0.1427 (4) 0.2234 (4)	374.393 (3) 0.1775 (2) 0.2072 (3)	374.384 (2) 0.1302 (6) 0.1708 (2)	373.629 (1) 0.1329 (5) 0.1552 (1)
German pertussis PT IgG (44:1987)	379.477 (6) 0.1891 (6) 2.1025 (6)	378.741 (5) 0.1906 (5) 0.5796 (5)	376.344 (3) 0.2837 (3) 0.2250 (3)	377.239 (4) 0.2622 (4) 0.2296 (4)	373.950 (2) 0.5100 (2) 0.1748 (2)	373.595 (1) 0.5233 (1) 0.1530 (1)
German pertussis PRN IgG (44:1992)	381.059 (2) 0.8700 (1) 0.4318 (6)	381.081 (4) 0.8619 (2) 0.3044 (5)	381.124 (6) 0.8263 (4) 0.1855 (4)	381.028 (1) 0.8560 (3) 0.1819 (3)	381.080 (3) 0.8141 (5) 0.1719 (2)	381.087 (5) 0.7829 (6) 0.1514 (1)
German pertussis FIM IgG (44:1986)	376.847 (6) 0.8273 (1) 0.5638 (6)	376.683 (5) 0.8039 (3) 0.4063 (5)	376.553 (3) 0.7381 (4) 0.2784 (4)	376.080 (1) 0.8222 (2) 0.2771 (3)	376.640 (4) 0.7028 (5) 0.2288 (2)	376.518 (2) 0.6791 (6) 0.1936 (1)
German pertussis FHA IgA (44:1932)	418.378 (4) 0.7157 (4) 0.1317 (1)	-	417.617 (3) 0.7216 (3) 0.1490 (4)	-	417.604 (2) 0.7217 (1) 0.1490 (3)	417.603 (1) 0.7217 (2) 0.1490 (2)
German pertussis PT IgA (44:1933)	418.782 (5) 0.5103 (1) 0.2860 (4)	418.784 (6) 0.5099 (2) 0.3207 (5)	418.101 (3) 0.4777 (6) 0.1490 (3)	418.751 (4) 0.5042 (3) 0.3621 (6)	418.076 (2) 0.4781 (5) 0.1490 (2)	418.075 (1) 0.4781 (4) 0.1490 (1)
German pertussis PRN IgA (44:1968)	-	-	406.932 (3) 0.2489 (3) 0.1490 (3)	-	406.479 (2) 0.2528 (2) 0.1489 (2)	406.432 (1) 0.2532 (1) 0.1488 (1)
German pertussis FIM IgA (44:1994)	410.398 (6) 0.0107 (5) 0.8372 (6)	410.361 (5) 0.0113 (3) 0.7657 (5)	410.349 (4) 0.0118 (2) 0.5205 (4)	409.896 (3) 0.0109 (4) 0.2282 (3)	408.899 (2) 0.0063 (6) 0.1776 (2)	407.798 (1) 0.0196 (1) 0.1721 (1)
Piedra RSV/A (34:175)	-	-	159.291 (3) 0.6080 (4) 0.9513 (4)	159.477 (4) 0.6156 (3) 0.4410 (3)	158.612 (2) 0.6694 (2) 0.2956 (2)	157.895 (1) 0.7361 (1) 0.2119 (1)
Piedra RSV/B (34:175)	-	-	154.415 (2) 0.6934 (3) 0.7328 (3)	-	154.395 (1) 0.7439 (2) 0.3734 (2)	154.553 (3) 0.7591 (1) 0.2643 (1)
White/varicella (79:3459)	643.035 (4) 0.9848 (5) 0.2887 (6)	642.145 (3) 0.9987 (3) 0.2297 (5)	641.683 (1) 1.0000 (1) 0.1802 (3)	641.765 (2) 1.0000 (2) 0.2000 (4)	643.060 (5) 0.9934 (4) 0.1403 (2)	643.507 (6) 0.9841 (6) 0.1314 (1)
Swedish pertussis FHA IgG (92:209)	-	-	284.404 (1) 0.0100 (1) 0.0777 (1)	-	-	-
Swedish pertussis PT IgG (92:209)	270.365 (6) 0.9223 (6) 0.1577 (4)	270.205 (5) 0.9274 (5) 0.1587 (5)	269.832 (4) 0.9379 (4) 0.1483 (3)	269.757 (3) 0.9407 (3) 0.1938 (6)	269.374 (2) 0.9529 (2) 0.1341 (2)	268.871 (1) 0.9754 (1) 0.1216 (1)
Swedish pertussis PRN IgG (92:209)	247.593 (5) 0.7605 (6) 0.0807 (4)	247.621 (6) 0.7700 (5) 0.0813 (5)	247.571 (4) 0.8123 (1) 0.0813 (6)	247.304 (3) 0.7736 (4) 0.0791 (3)	246.989 (2) 0.7884 (2) 0.0772 (2)	246.071 (1) 0.7863 (3) 0.0763 (1)
Swedish pertussis FIM IgG (92:209)	251.075 (6) 0.2775 (6) 0.3644 (6)	250.822 (5) 0.2921 (5) 0.2708 (5)	250.161 (4) 0.3250 (4) 0.1697 (4)	249.765 (3) 0.3530 (3) 0.1470 (3)	248.902 (2) 0.3691 (2) 0.1103 (2)	247.731 (1) 0.4765 (1) 0.0941 (1)
Black Nicolay HAI (22:777)	181.035 (6) 0.5029 (6) 0.2875 (6)	180.649 (5) 0.5241 (4) 0.2661 (5)	179.402 (3) 0.5209 (5) 0.2267 (3)	179.675 (4) 0.5886 (2) 0.2329 (4)	177.247 (2) 0.5479 (3) 0.2122 (2)	176.743 (1) 0.6813 (1) 0.2120 (1)

MLEs were found for 81 of the 96 combinations of protection curve function and illustrative dataset.

There were differences between models with different protection functions. For nine of the sixteen datasets the difference between the smallest and largest −2×log-likelihood exceeded 1; for one it exceeded 5. For eleven datasets, models with the absolute sigmoid protection function had the smallest −2×log-likelihood. Models with this protection function also had the best goodness-of-fit for eight datasets and the smallest coefficient of variation of $\widehat{\lambda}$ for fourteen datasets.

Some datasets had consistently good goodness-of-fit with all protection functions; for example, for the White/varicella dataset it ranged from 0.984 to 1.000, for the Swedish pertussis PT dataset from 0.922 to 0.975. For others it was consistently poor, not exceeding 0.020 for any protection function with the German pertussis FIM IgA dataset. For some datasets, goodness-of-fit was more variable, e.g. the German pertussis PT IgG dataset. Coefficients of variation of $\widehat{\lambda}$ typically ranged from 0.15 to 0.3.

In general, models with protection functions which approached the asymptotes more slowly found more MLEs and had better evaluation criteria than models with protection functions which approached the asymptotes more quickly.

### Generalized symmetrical protection curve

For seven datasets a model with the generalized symmetrical protection curve substantially improved evaluation criteria relative to the model with the optimal 2-parameter protection curve. Among these datasets, reductions in −2×log-likelihood from more than 1 to more than 16 were seen, and for most of them improvement in goodness-of-fit was equally marked. For other datasets, improvement was more modest or no improvement was seen. Detailed results for models with the generalized symmetrical protection curve are given in Additional file 4.

### ‘Incomplete protection’ protection curves

For nine of the eleven datasets for which MLEs were found, −2×log-likelihood of the optimal ‘incomplete protection’ model was at least 1 smaller than for the optimal 2-parameter model. Of particular note was the Swedish pertussis FHA IgG dataset, for which MLEs were found with only one of the 2-parameter models; when ‘incomplete protection’ protection curves were used MLEs were found with all six models. Marked consistency in likelihood and fit between incomplete protection models was noted, whichever 2-parameter function was used.

Detailed results of fitting models with ‘incomplete protection’ protection curves are given in Additional file 4.

### Non-symmetrical protection curves

Of the four non-symmetrical models evaluated, none was consistently optimal. Notably, for the Swedish pertussis FHA IgG dataset, for which MLEs were found with only one of the 2-parameter symmetrical models, MLEs were found with three of the four asymmetrical models. In 25 out of a possible 64 instances, a non-symmetrical model was an improvement over the corresponding symmetrical model, either finding MLEs where the symmetrical model did not, or improving on all three evaluation criteria. In most instances the improvement was small, though in eleven instances −2×log-likelihood decreased by more than 1. For three datasets, −2×log-likelihood and goodness-of-fit for the non-symmetrical models were almost identical to the symmetrical models. Detailed results for models with non-symmetrical protection curves are given in Additional file 1.

### Case-cohort designs

Among the 57 combinations of illustrative dataset, protection curve and non-case:case ratio assessed, MLEs were found for all 100 case-cohort datasets 43 times. Among the remaining 14 combinations, MLEs were found for between 45 and 99 of the case-cohort datasets; however for the error function model fitted to the German pertussis FHA IgA case-cohort datasets the number of MLEs did not exceed 50 for any of the three values of non-case:case ratio. For the combinations where less than 100 MLEs were found there was an observable trend towards more MLEs being found when the non-case:case ratio was higher. Detailed results for the case-cohort datasets are given in Additional file 5.

Similarly, there was a visible trend for the median standard error of the estimates of the location parameter, SE($\widehat{\alpha}$), to increase as the non-case:case ratio decreased. When the non-case:case ratio was 20 the median value of SE($\widehat{\alpha}$) in the case-cohort datasets was close to its value in the ‘all-subjects’ dataset; when the non-case:case ratio was 5 the median value of SE($\widehat{\alpha}$) in the case-cohort datasets was typically 15 % to 40 % greater than in the ‘all-subjects’ dataset.

### Standard errors and confidence intervals

Among the 54 combinations of protection function, illustrative dataset and bootstrap method assessed, MLEs were found for 46,591 of the 54,000 bootstrap datasets – 17,055 out of 24,000 with error function models and 29,536 out of 30,000 with absolute sigmoid models. Detailed results for the estimates of standard errors are given in Additional file 6.

There was considerable variability in the estimates of the standard error of $\widehat{\alpha}$ based on the observed information. Among the 54 combinations, there were six where more than 50 % of the estimates were less than half or more than twice their median. Among the non-parametric bootstrap datasets there were only two combinations where more than 90 % of the estimates were between half and twice the median; among the parametric bootstraps there was only one where more than 90 % of the estimates were between half and twice the median.

The median standard error of $\widehat{\alpha}$ calculated from the observed information was less than the standard deviation of the bootstrap estimates of $\widehat{\alpha}$, $SD\left(\widehat{\alpha}\right)$, for 45 out of the 54 combinations – 16 out of 24 for the error function models and 29 out of 30 for the absolute sigmoid models. The difference was particularly marked for the absolute sigmoid models, where there were combinations where the median was 1/5^th^ (three instances), 1/7^th^, 1/13^th^ 1/16^th^ and 1/22^nd^ the value of $SD\left(\widehat{\alpha}\right)$.

The standard deviation of the bootstrap estimates of $\widehat{\alpha}$, $SD\left(\widehat{\alpha}\right)$, was consistently smaller in the absolute sigmoid models than in the error function models.

The coverage probability of 95 % two-sided confidence intervals for *α* , calculated from the observed information by $\widehat{\alpha}\pm 1.96\times SE\left(\widehat{\alpha}\right)$, in the parametric bootstrap datasets, is shown in Table 2.

**Table 2:** Coverage probability of 95 % two-sided confidence intervals for *α* , calculated from the observed information by $\widehat{\alpha}\pm 1.96\times SE\left(\widehat{\alpha}\right)$, in the parametric bootstrap datasets

Dataset (cases of disease:subjects)	Error function models		Absolute sigmoid models	
	N*	Coverage	N*	Coverage
German pertussis FHA IgG (44:1988)	801	0.925	1000	0.642
German pertussis PT IgG (44:1987)	595	0.797	1000	0.658
German pertussis PRN IgG (44:1992)	826	0.924	1000	0.619
German pertussis FIM IgG (44:1986)	479	0.987	1000	0.711
German pertussis FHA IgA (44:1932)	194	0.866	1000	0.216
German pertussis PT IgA (44:1933)	232	0.991	1000	0.225
German pertussis PRN IgA (44:1968)	-	-	1000	0.320
German pertussis FIM IgA (44:1994)	656	0.942	999	0.650
Piedra RSV/A (34:175)	-	-	964	0.773
Piedra RSV/B (34:175)	-	-	877	0.814
White/Varicella (79:3459)	998	0.921	1000	0.941
Swedish pertussis FHA IgG (92:209)	-	-	-	-
Swedish pertussis PT IgG (92:209)	846	0.953	969	0.829
Swedish pertussis PRN IgG (92:209)	951	0.930	1000	0.744
Swedish pertussis FIM IgG (92:209)	697	0.811	991	0.772
Black Nicolay HAI (22:777)	790	0.934	1000	0.614

* number of bootstrap datasets for which MLEs were found

Although with the error function model the coverage probability was more than 90 % for all but two illustrative datasets, it exceeded this percentage for only one of the fifteen illustrative datasets with the absolute sigmoid model.

The proportion of subjects with assay values above or below the 95 % confidence interval for the value at which protection was 50 %, using the mean of the standard deviation of $\widehat{\alpha}$ from parametric and non-parametric bootstraps for the standard error, was greater than 80 % for seven of the fifteen illustrative datasets with absolute sigmoid models, and less than 40 % for only one such combination; for error function models, the proportion of subjects with assay values outside the confidence interval was greater than 80 % for only one of the twelve datasets, and was less than 40 % for seven.

## Discussion

An important finding was that there was considerable variability in the estimated standard error of the location parameter based on the observed information in both parametric and non-parametric bootstraps. In addition, the median standard error based on the observed information was markedly less than the standard deviation of bootstrap parameter estimates; this was particularly pronounced for the absolute sigmoid model, which was generally the best-fitting of the 2-parameter symmetric curve models. Further, the coverage probability of confidence intervals based on the observed information was poor, again particularly for the absolute sigmoid model. Although confidence intervals based on observed information are unbiased asymptotically [37], it appeared that for the models investigated here fitted to the datasets used for illustration, and particularly for a better fitting model, they could not be relied upon. Of the methods considered bootstrap confidence intervals were to be preferred.

Reliable estimates of precision for immune correlates of protection are necessary for proper interpretation of results. For example, Plotkin notes different authors have found point estimates for HAI titers of 1:40, 1:15, 1:30, 1:110, 1:32 and 1:64 to be protective against clinical influenza [38]; without knowledge of the precision of the estimates it cannot be inferred whether these differences represent true differences in the circumstances of each experiment or are simply due to chance.

Reliable confidence intervals for measures of protection are also necessary for valid classification of individuals as susceptible or protected, and for assessment of the utility of results. Principles of statistical inference dictate that only individuals with assay values below the lower limit of the confidence interval can confidently be said to be susceptible by the measure, and only those above the upper limit may confidently be said to be protected. To suggest that susceptibility or protection can be determined by reference to the point estimate departs from accepted conventions of inference.

The utility of an estimate of a measure of protection may be assessed by comparing its confidence interval with the distribution of subjects’ assay values; an estimate for which most subjects’ assay values fell within the confidence interval would have little utility. A reasonable standard of precision for a measure might be one that had been shown to classify 85 % of subjects as either susceptible or protected with 95 % confidence. In the two models investigated, this was achieved for six of the fifteen illustrative datasets for which MLEs were found with the better-fitting model, but only one of the twelve datasets with the less well-fitting model.

Of the models investigated, no model was uniformly optimal for all datasets. For every illustrative datasets, there was at least one 2-parameter symmetrical model which improved both likelihood and goodness-of-fit relative to the logistic (scaled logit) model. There was also at least one ‘additional parameter’ model – generalized symmetric, incomplete protection or asymmetric – which improved likelihood over the best 2-parameter model, and goodness-of-fit was likewise improved for all but one dataset. However, the degree of improvement was variable, ranging from 0.101 in −2×log-likelihood and no improvement in fit (White/varicella dataset) to 16.597 improvement in −2×log-likelihood and 0.947 in goodness-of-fit (Swedish pertussis FHA dataset).

When the estimate of the shape parameter *κ* in the generalized symmetric model was between 1 and 3.3 there was (as would be expected) a 2-parameter model with closely similar likelihood and goodness-of-fit, and variability in likelihood and goodness-of-fit was limited across other 2-parameter models (three datasets). For two datasets with shape parameter estimates of 9.0 and 16.7, the 2-parameter absolute sigmoid model had similar likelihood, and variability in likelihood and fit were also limited. When the estimate of the shape parameter in the generalized symmetric model was less than or equal to 0.3 and the fit good (>0.69), there was an incomplete protection model with closely similar likelihood and goodness-of-fit; as between the two the incomplete protection model would be preferred as better estimating exposure (6 datasets).

For three datasets goodness-of-fit was poor, less than 0.3, for all models; for one (German pertussis FIM IgG) likelihood and goodness-of-fit were variable and inconsistent; and for one (Swedish pertussis PT) goodness-of-fit was excellent for all models with which MLEs were found, though the generalized symmetric model was not among them.

A reasonable strategy for an exploratory analysis of a dataset, based on the results for these illustrative datasets, might be to first attempt to fit a generalized symmetric model. An estimate of the shape parameter *κ* between 1 and 3.3 would suggest fitting the corresponding 2-parameter protection curve, which, other things being equal, would be preferred for parsimony. Small values of *κ*, less than 0.3, would suggests an incomplete protection model, possibly using the logistic function since this is the canonical link function for binary data. Larger values of *κ*, might suggest an absolute sigmoid model.

However, this strategy would not have found the optimal model for the Swedish pertussis PT dataset.

An alternative could be to first attempt an absolute sigmoid model, since this ranked favorably among the 2-parameter models for many datasets. Another alternative, used by one of the authors, is to note than many elements of the computer code required for an analysis are common to most models – capturing parameter estimates, −2×log-likelihood, the eigenvalues, calculating the goodness-of-fit – and having written code for these a variety of models can be investigated with minimal additional effort.

For some datasets, spline models had better likelihood and goodness-of-fit than other models. However, it was noted that the fitting algorithm often began to fail to find MLEs at it approached what appeared to be the maximum of the profile likelihood, and the spline results for a number of datasets reflect the greatest likelihood among the knots for which MLEs were found, rather than a global maximum.

For the multiple datasets used for illustration here, goodness-of-fit and −2×log-likelihood were used to compare models. For a single datasets, plots of fitted curves and protection curves, consideration of whether parameter estimates are bounded, proportions of bootstrap datasets for which MLEs are found, and stability of estimates obtained from different starting values or when convergence criteria are tightened, can also contribute to model selection and evaluation.

Case-cohort designs appeared to result in little loss of precision when the ratio of non-cases to cases was 20:1; such a design would have reduced the number of samples assayed by approximately half for the three studies (ten datasets) investigated. A small loss of precision was apparent when the ratio was 5:1; such a ratio would have reduced the number of samples assayed by 90 %.

We have shown that there are a number of alternatives to the logistic protection curve proposed in the scaled logit model that improve the fit of the model and the precision of estimates. The investigations did, however, reveal the challenges involved in estimating the relationship between two variables, assay value and disease, from datasets powered to estimate a single-valued endpoint such as vaccine efficacy – in the areas of achieving convergence and finding MLEs, achieving convergence to a consistent point, variance estimation, achieving a reasonable standard of precision, and sometimes marked differences in fit and precision between closely similar models – particularly in the multi-parameter non-linear models which are needed to explicitly quantify protection. The authors’ experience has been that with richer, more informative simulated datasets many of these challenges disappear; however, it is unrealistic to expect that larger, more informative designed experiments will often be conducted; cost and ethical considerations would be difficult to surmount.

We gave no consideration to the question of adjusting for multiple comparisons, and inference from fitting multiple models cannot be made to a population. We applied the same fitting criteria (for convergence, positive definite Hessian and starting values) in all evaluations without consideration of whether they were uniformly applicable to all datasets.

Further research might consider improved methods for estimating variability; non-parametric methods for estimating protection curves might also be explored. The protection afforded vaccinees may not be the same as for non-vaccinees with the same assay value, and previous research has suggested models separately estimating protection in different treatment groups [24, 25]. Even when the Prentice criterion is considered met or the proportion of treatment explained is high, a common protection curve does not predict the observed relative risk and hence is not predictive of vaccine efficacy; methods predictive of efficacy would be of interest. When more than one assay is conducted, methods for combining their data to estimate protection would be valuable. Other approaches proposed to estimate correlates of protection, such as the method of Chang and Kohberger [17, 39], the a:b model [19], the vaccine efficacy curve method [3, 14, 16], and area-under-the-curve methods [21, 40] might be compared and reconciled with those suggested here.

## Conclusions

The original scaled logit model can be improved upon by considering alternative protection curve functions and refining likelihood maximization methods. Bootstrap methods for estimating precision are to be preferred over estimates based on the observed information. Evaluation criteria permit well-fitting models and useful results to be identified. With some datasets good model fit and precision can be achieved with simpler models; others require more complex models. The proposed methods provide a comprehensive set of tools for quantifying the relationship between immunological assays and protection from disease.

## Additional files

Additional file 1:
**Detail of methods and results for non-symmetrical protection curve models.** (DOCX 116 kb) Additional file 2:
**Detail for limiting the steepness of the protection curve, maximum likelihood estimates, evaluation criteria and fitting and calculation notes.** (DOCX 47 kb) Additional file 3:
**Illustrative SAS code to create the datasets reconstructed from published sources and fit models.** (DOCX 50 kb) Additional file 4:
**Detail of results of fitting models with generalized symmetrical protection curve and with ‘incomplete protection’ protection curves.** (DOCX 84 kb) Additional file 5:
**Detail of results of case-cohort investigation.** (DOCX 41 kb) Additional file 6:
**Detail of results for estimation of standard errors.** (DOCX 59 kb)
