# Supplementary material for: Some extensions in continuous models for immunological correlates of protection
Source: BMC Med Res Methodol. 2015 Dec 28;15:107. doi: 10.1186/s12874-015-0096-9 (PMC4692073; doi:10.1186/s12874-015-0096-9)
Supplement: Additional file 1: — Detail of methods and results for non-symmetrical protection curve models. (DOCX 116 kb) [file 12874_2015_96_MOESM1_ESM.docx]

**Additional file 1: Detail of methods and results for non-symmetrical protection curve models**

*Illustration of* $t_{\nu}=t_{N}^{\nu}\log\left(t_{N}\right)$ *transformation*

The effect of the transformation for different values of *ν* is illustrated in the following figure; transformed values have been rescaled to bring them all into the interval [0,1] for comparison.

*Methods for2-part splined curves*

A two-part splined model consists of the lower left and upper right parts of two curves with different exposure, location and slope parameters constrained to join smoothly at a ‘knot’. A general form is

$$\mathrm{P}\left(\text{disease}\right)=1\left(t<t_{knot}\right) \lambda_{1}\left[1-\pi\left(\beta_{1}\left(t-\alpha_{1}\right)\right)\right]+1\left(t>t_{knot}\right)\lambda_{2}\left[1-\pi\left(\beta_{2}\left(t-\alpha_{2}\right)\right)\right]$$

subject to the constraints

$\lambda_{1}\left[1-\pi\left(\beta_{1}\left(t_{knot}-\alpha_{1}\right)\right)\right]= \lambda_{2}\left[1-\pi\left(\beta_{2}\left(t_{knot}-\alpha_{2}\right)\right)\right]$ [1]

 [2]

where subscripts ⋅_1_ and ⋅_2_ refer to the lower left and upper right parts of the protection curve respectively and 1(⋅) is the indicator function taking the value 1 when its argument is true and 0 when it is false. The two parts of the curve join smoothly at a ‘knot’, *t_knot_*.

There are seven parameters and two constraints, leaving five parameters to be estimated.

Since the likelihood is not continuous in the *t_knot_* parameter, a profile likelihood is constructed over candidate values of *t_knot_* and the maximum chosen. The candidate values of *t_knot_* may be taken to be the means of adjacent pairs of the ordered observed values of *t*, and the model is fitted by proceeding through the candidate values of *t_knot_*, at each value calculating two parameters and maximizing the likelihood across the remaining four, and then selecting the value of *t_knot_* which maximizes the likelihood overall.

Dividing [1] by [2] shows that there is a functional relationship between *α*_1_, *α*_2_, *β*_1_ and *β*_2_, so the likelihood cannot be maximized over this space. Solving the spline equations for *α*_1_ and *α*_2_ and maximizing the likelihood over *λ*_1_, *λ*_2_, *β*_1_ and *β*_2_ allows the *β* s to be bounded; however, inverting the protection function is required. Some 2-parameter protection functions are algebraically invertible and some are not; simplest was found to solve [2] for *α*_1_ or *α*_2_, and then find the value of the other *α* which solves [1] numerically, which will be possible because *π* (⋅) is monotone in *α*.

For the error function (i.e. with *π* (⋅) being the cumulative normal probability function Φ (⋅)), [2] gives

$$\alpha_{2}= t_{knot}\pm\frac{1}{\beta_{2}}\sqrt{\left(\left(\beta_{1}\left(t_{knot}-\alpha_{1}\right)\right)^{2}-2\log\left(\frac{\lambda_{1}\beta_{1}}{\lambda_{2}\beta_{2}}\right)\right)}$$

and when$\lambda_{2}\beta_{2}>\lambda_{1}\beta_{1}$ the argument to the square root will always be positive. For the sign, ±, it was found that the sign of *λ*_1_−*λ*_2_ most frequently found MLEs. Thus an algorithm to find numerical solutions for *α*_1_ and *α*_2_ is as follows.

*i*=0;

if *λ*_2_*β*_2_>*λ*_1_*β*_1_ then do;

*α*_10_=*t_knot_*×0.999;

*α*_1_= *t_knot_*×0.9999;

*α*_2_= *t_knot_* + sign(*λ*_1_−*λ*_2_) sqrt((*β*_1_(*t_knot_*−*α*_10_))^2^−2log(*λ*_1_*β*_1_/(*λ*_2_*β*_2_))) /*β*_2_;

*δ*_0_= *λ*_1_(1−Φ(*β*_1_(*t_knot_*−*α*_10_))) − *λ*_2_(1−Φ(*β*_2_(*t_knot_*−*α*_2_)));

do until ((abs(*δ*)<10^−9^) or (*i* >100));

*i*= *i*+1;

*α*_2_= *t_knot_* + sign(*λ*_1_−*λ*_2_) sqrt((*β*_1_(*t_knot_*−*α*_1_))^2^−2log(*λ*_1_*β*_1_/(*λ*_2_*β*_2_))) /*β*_2_;

*δ*= *λ*_1_(1−Φ(*β*_1_(*t_knot_*−*α*_1_)))− *λ*_2_(1−Φ(*β*_2_(*t_knot_*−*α*_2_)));

*α*_12_= *α*_1_ − *δ* (*α*_1_−*α*_10_)/(*δ*−*δ*_0_);

*α*_10_=*α*_1_;

*α*_1_=*α*_12_;

*δ*_0_=*δ*;

end;

end;

else do;

*α*_20_= *t_knot_*×1.001;

*α*_2_= *t_knot_*×1.0001;

*α*_1_= *t_knot_* + sign(*λ*_2_−*λ*_1_) sqrt( (*β*_2_(*t_knot_*−*α*_20_))^2^−2log(*λ*_2_*β*_2_/(*λ*_1_*β*_1_)))/*β*_1_;

*δ*_0_= *λ*_2_(1−Φ(*β*_2_(*t_knot_*−*α*_20_))) − *λ*_1_(1−Φ(*β*_1_(*t_knot_*−*α*_1_)));

do until ((abs(*δ*)<10^−9^) or (*i* >100));

*i*= *i*+1;

*α*_1_= *t_knot_* + sign(*λ*_2_−*λ*_1_) sqrt( (*β*_2_(*t_knot_*−*α*_2_))^2^−2log(*λ*_2_*β*_2_/(*λ*_1_*β*_1_)))/*β*_1_;

*δ*= *λ*_2_(1−Φ(*β*_2_(*t_knot_*−*α*_2_))) − *λ*_1_(1−Φ(*β*_1_(*t_knot_*−*α*_1_)));

*α*_22_= *α*_2_ − *δ* (*α*_2_−*α*_20_)/(*δ*−*δ*_0_);

*α*_20_=*α*_2_;

*α*_2_=*α*_22_;

*δ*_0_=*δ*;

end;

end;

For the absolute sigmoid two-spline model, [2] gives

$$\left|\beta_{2}\left(t_{knot}-\alpha_{2}\right)\right|= \pm\sqrt{\frac{\lambda_{2}\beta_{2}}{\lambda_{1}\beta_{1}}}\left(1+\left|\beta_{1}\left(t_{knot}-\alpha_{1}\right)\right|\right)-1$$

For this to be generally true requires the positive sign be taken and$\lambda_{2}\beta_{2}>\lambda_{1}\beta_{1}$. Assuming *β*_2_>0 (it can be bounded to be so in the maximization algorithm) yields

$$\alpha_{2}= t_{knot}\pm\frac{1}{\beta_{2}}\left(\sqrt{\frac{\lambda_{2}\beta_{2}}{\lambda_{1}\beta_{1}}}\left(1+\left|\beta_{1}\left(t_{knot}-\alpha_{1}\right)\right|\right)-1\right)$$

and thus solutions may be found for *α*_1_ and *α*_2_ in a similar manner as for the error function and the likelihood over *λ*_1_, *λ*_2_, *β*_1_ and *β*_2_ calculated and maximized.

For splined models the *β* s were bounded as previously described and *λ*_2_ bounded 0<*λ*_2_<5. MLEs were defined as previously and in addition fittings which estimated protection curves with three inflection points, *α*_1_<*t_knot_*<*α*_2_, were excluded.

*Results*

The evaluation criteria from fitting the four non-symmetrical models are shown in the following table. The coefficients of variation of the spline models are conditional on the knots selected and hence not strictly comparable with those of the other models.

| Parameter estimate −2×log‑likelihood  Goodness-of-fit Coef. of Var. $\hat{\lambda}$ | Protection curve/function | | | |
| --- | --- | --- | --- | --- |
| Dataset (cases of disease:subjects) | Error function with *t_ν_*=*t*_N_*^ν^*log(*t*_N_) | Error function two‑part spline | Absolute sigmoid with *t_ν_*=*t*_N_*^ν^*log(*t*_N_) | Absolute sigmoid two‑part spline |
| German pertussis FHA IgG (44:1988) | - - - | 373.438 0.2369 0.1626 | $\hat{\nu}=$ 0.226 373.486 0.1566 0.2507 | 372.724 0.1273 0.1619 |
| German pertussis PT IgG (44:1987) | - - - | 378.682 0.2641 0.1999 | $\hat{\nu}=$−0.118 373.257 0.5724 0.1508 | 373.121 0.7236 0.1577 |
| German pertussis PRN IgG (44:1992) | $\hat{\nu}=$ 0.002 381.059 0.8702 0.9095 | 380.939 0.8680 0.2106 | $\hat{\nu}=$ 0.161 381.051 0.7866 0.2046 | 381.159 0.8312 0.1569 |
| German pertussis FIM IgG (44:1986) | - - - | 375.156 0.8918 0.1888 | $\hat{\nu}=$ 0.376 385.501 0.0608 0.1482 | 374.954 0.6205 0.1647 |
| German pertussis FHA IgA (44:1932) | - - - | 417.592 0.7207 0.1532 | $\hat{\nu}=$ 0.453 417.532 0.7222 0.1490 | 417.605 0.7217 0.1608 |
| German pertussis PT IgA (44:1933) | - - - | 417.959 0.4953 1.6362 | $\hat{\nu}=$ 0.764 417.956 0.4805 0.1490 | 418.071 0.4799 0.1380 |
| German pertussis PRN IgA (44:1968) | - - - | 407.579 0.2413 0.1496 | $\hat{\nu}=$ 1.034 405.990 0.2557 0.1487 | 406.447 0.2532 - |
| German pertussis FIM IgA (44:1994) | $\hat{\nu}=$−0.122 410.315 0.0115 0.7180 | 410.007 0.0110 0.3409 | - - - | 406.153 0.0261 0.1619 |
| Piedra RSV/A  (34:175) | - - - | 160.091 0.5899 0.2104 | - - - | 157.995 0.7546 0.2010 |
| Piedra RSV/B  (34:175) | $\hat{\nu}=$−0.103 154.232 0.7844 0.4637 | 171.319 0.0027 0.1558 | $\hat{\nu}=$ 0.048 154.537 0.7412 0.5061 | 154.774 0.7527 0.2070 |
| White/Varicella  (79:3459) | $\hat{\nu}=$−0.187 641.864 1.0000 0.2300 | 642.406 0.9998 0.1631 | $\hat{\nu}=$ 0.638 642.359 0.9997 0.2854 | 645.851 0.8925 0.1324 |
| Swedish pertussis FHA IgG (92:209) | - - - | 284.402 0.0100 0.0777 | $\hat{\nu}=$−0.168 268.638 0.9348 0.1685 | 269.392 0.8877 0.1502 |
| Swedish pertussis PT IgG (92:209) | - - - | 269.343 0.9633 0.0895 | - - - | 268.216 0.9902 0.0790 |
| Swedish pertussis PRN IgG (92:209) | $\hat{\nu}=$−0.090 247.471 0.8396 0.0827 | 246.958 0.5681 0.0724 | - - - | 244.622 0.8149 0.0710 |
| Swedish pertussis FIM IgG (92:209) | - - - | 249.774 0.2677 0.0727 | - - - | 245.989 0.5210 0.0709 |
| Black Nicolay HAI (22:777) | - - - | 179.373 0.6347 0.2230 | - - - | 175.904 0.7743 0.2176 |

For two-part spline models the protection curve is estimated from the fitted model by. By way of illustration, the estimated protection curve for the absolute sigmoid two-part spline model fitted to the Swedish pertussis PT IgG data is shown in the following figure.
