# Supplementary material for: Some extensions in continuous models for immunological correlates of protection
Source: BMC Med Res Methodol. 2015 Dec 28;15:107. doi: 10.1186/s12874-015-0096-9 (PMC4692073; doi:10.1186/s12874-015-0096-9)
Supplement: Additional file 2: — Detail for limiting the steepness of the protection curve, maximum likelihood estimates, evaluation criteria and fitting and calculation notes. (DOCX 47 kb) [file 12874_2015_96_MOESM2_ESM.docx]

**Additional file 2: Detail for limiting the steepness of the protection curve, maximum likelihood estimates, evaluation criteria and fitting and calculation notes**

The likelihood maximization algorithm can fail to converge if the steepness of the protection curve increases until the slope parameter exceeds computer limits, i.e. the protection curve approaches a step function. This may be prevented by bounding the slope parameter in the maximization algorithm. A reasonable bound might be one where protection increased from 1% to 99% in some small fraction of the range of the assay values, say 1/50^th^; the curve would then be virtually indistinguishable from a step function. Such a bound would be

*β* ≤ 2π^−1^(0.99) × 50 ÷ range(log-assay value)

for symmetrical curves or *β* ≤ [π^−1^(0.99) − π^−1^(0.01)] × 50 ÷ range(log-assay value) more generally, where π^−1^(⋅) is the inverse of the protection function. The values of $2\pi^{-1}(0.99)$for the 2-parameter symmetrical protection functions are:

| error function | 4.6527 |
| --- | --- |
| logistic function | 9.1902 |
| square root sigmoid function | 9.8494 |
| double exponential function | 7.8240 |
| arctangent function | 63.641 |
| absolute sigmoid function | 98 |

For the generalized symmetric protection function, where π^−1^(⋅) is also a function of *κ*, *β* was bounded at 10^12^.

It was found that with the default value of the relative gradient convergence criterion in the maximization algorithm used (SAS *proc nlmixed*; *gconv* = 10^−8^) different starting values converged to different MLEs, and the criterion was therefore tightened (to 10^−12^) and models with different starting values fitted. It was noted that occasionally different starting values converged to close but different points even with tightened convergence criteria.

For fittings other than bootstraps and splines seven sets of starting values were used

| ‘standard’ | *λ* = 2 × mean rate of disease  *α* = midpoint(log-assay value) *β* = 2π^−1^(0.99) ÷ range(log‑assay value) |
| --- | --- |
| ‘high λ’ | *λ* = min(3.2 × mean rate of disease, 0.9)  *α* = midpoint(log-assay value) *β* = 2π^−1^(0.99) ÷ range(log‑assay value) |
| ‘low λ’ | *λ* = 1.2 × mean rate of disease  *α* = midpoint(log-assay value) *β* = 2π^−1^(0.99) ÷ range(log‑assay value) |
| ‘high α’ | *λ* = 2 × mean rate of disease  *α* = midpoint(log-assay value) + 1 *β* = 2π^−1^(0.99) ÷ range(log‑assay value) |
| ‘low α’ | *λ* = 2 × mean rate of disease  *α* = midpoint(log-assay value) − 1 *β* = 2π^−1^(0.99) ÷ range(log‑assay value) |
| ‘high β’ | *λ* = 2 × mean rate of disease *α* = midpoint(log-assay value) *β* = 4π^−1^(0.99) ÷ range(log‑assay value) |
| ‘low β’ | *λ* = 2 × mean rate of disease  *α* = midpoint(log-assay value), and *β* = π^−1^(0.99) ÷ range(log‑assay value) |

For fitting bootstrap datasets two sets of starting values were used – ‘standard’, and the parameter estimates from the illustrative dataset. For fitting splines, parameter estimates from the last knot at which MLEs were found were used, followed by a set of values approximating knots at which MLEs had frequently been found, followed by ‘standard’ values, until MLEs were found.

The Hessian matrix was considered positive definite if all eigenvalues were greater than −10^−4^, since this found a large number of MLEs which would have been excluded if the condition had been all eigenvalues greater than 0.

For incomplete protection curve models, the incomplete protection parameter *γ* was required to be in (0,1).

In models with symmetrical protection curves and the $\pi\left(t;\alpha,\beta,...\right)=\pi\left(\beta\left(t-\alpha\right),...\right)$ parameterization *α* is the log-assay value at which protection is 50%. For other models and percentages the protection function must be inverted to estimate the assay value at which protection is a certain percentage; for example, the log-assay value at which protection is 80% is given by. While it is possible to invert some functions algebraically, and numerical inversion is always possible since the protection function is monotone, a good approximation to the assay value at which protection is 50% or any other percentage may be found by interpolation of the estimated protection curve derived from the fitted values returned by the maximization algorithm, and was used routinely.
