# Supplementary material for: Some extensions in continuous models for immunological correlates of protection
Source: BMC Med Res Methodol. 2015 Dec 28;15:107. doi: 10.1186/s12874-015-0096-9 (PMC4692073; doi:10.1186/s12874-015-0096-9)
Supplement: Additional file 3: — Illustrative SAS code to create the datasets reconstructed from published sources and fit models. (DOCX 50 kb) [file 12874_2015_96_MOESM3_ESM.docx]

**Additional file 3: Illustrative SAS code to create the datasets reconstructed from published sources and fit models**

* Step 1: create dataset;

* Step 1, example 1: create White/varicella dataset;

* from Table 1 of White CJ, Kuter BJ, et al. Modified cases of chickenpox after varicella

vaccination: correlation of protection with antibody response. Pediatric Infectious Disease

Journal 1992, 11:19-23;

**data** White0;

infile datalines delimiter=',';

input low high N pcnt_yr1;

datalines;

0.15, 0.29, 113, 11.8

0.3, 0.64, 55, 8.7

0.65, 1.3, 186, 8.8

1.4, 2.5, 239, 6.8

2.6, 4.9, 370, 3.4

5.0, 9.9, 617, 1.3

10.0, 19.9, 891, 0.6

20.0, 39.9, 988, 0.3

;

**data** corrprot; set White0;

do i= **1** to N; assayval_nat= sqrt(low*high); disease= (i le round(N*pcnt_yr1/**100**,**1**)); output; end;

keep assayval_nat disease; **run**;

* ... or ...;

* Step 1, example 2: create Black Nicolay HAI dataset;

* from Table 2 of Black S, Nicolay U, et al. Hemagglutination inhibition antibody titers as a

correlate of protection for inactivated influenza vaccines in children. Pediatric Infectious

Disease Journal 2011, 30(12):1081-5.;

**data** BlackNicolay0;

infile datalines delimiter=',';

input titer cases_plac n_plac cases_tiv n_tiv cases_adj n_adj;

datalines;

5,6,119,2,25,0,4

10,0,0,2,36,0,0

20,0,2,4,47,0,0

28,0,0,0,1,0,0

40,0,1,2,42,0,1

57,0,0,0,1,0,0

80,0,4,4,28,0,1

113,0,0,0,1,0,0

160,0,7,0,32,0,21

226,0,0,0,1,0,2

320,0,13,0,17,0,63

453,0,1,0,0,0,7

640,0,4,0,16,1,76

905,0,0,0,2,0,4

1280,0,0,0,35,1,63

1810,0,0,0,4,0,4

2560,0,1,0,19,0,46

3620,0,1,0,2,0,9

5120,0,0,0,4,0,10

;

**data** corrprot; set BlackNicolay0(rename= (titer=assayval_nat));

treat= 'Plac';

do i= **1** to cases_plac; disease=**1**; output; end;

do i= cases_plac+**1** to n_plac; disease=**0**; output; end;

treat= 'TIV';

do i= **1** to cases_TIV; disease=**1**; output; end;

do i= cases_TIV+**1** to n_TIV; disease=**0**; output; end;

treat= 'Adj';

do i= **1** to cases_Adj; disease=**1**; output; end;

do i= cases_Adj+**1** to n_Adj; disease=**0**; output; end;

keep assayval_nat disease treat; **run**;

* Step 2: submit the following macros and formats. The macros operate on a dataset named

corrprot, similar to that created in Step 1, containing at a minimum the variables

assayval_nat (assay value on the natural scale) and disease;

* Note re missing values/case-cohort designs: lines with missing values for disease are ignored, i.e.

it is assumed that the lines with non-missing disease represent a random sample of subjects from

the population of interest. If a there are missing values of assayval_nat it is assumed the dataset

comes from a case-cohort design;

**%macro** ***fit_model***;

%***prelim***;

%do startvals= **1** %to **7**;

%***fitmod***;

%end;

**%mend**;

**%macro** ***prelim***; * create working file and metadata;

data cp(keep= assayval_nat logassayval disease) metadata1(keep= protection_curve sample_size cases

minassayval_nat maxassayval_nat noncase_fract);

set corrprot(where= (not missing(disease))) end=eof;

retain minassayval_nat **10e12** maxassayval_nat -**10e12** disease_sum_all cases sample_size **0**;

protection_curve= symget('protection_curve');

disease_sum_all= disease_sum_all + disease;

if not missing(assayval_nat) then do;

sample_size= sample_size+**1**; logassayval= log(assayval_nat);

minassayval_nat= min(minassayval_nat,assayval_nat);

maxassayval_nat= max(maxassayval_nat,assayval_nat);

cases= cases + disease; output cp; end;

if eof then do;

noncase_fract= disease_sum_all*(sample_size-Cases)/Cases/(_n_-disease_sum_all); * non-case fraction

for case-cohort datasets; output metadata1; end; run;

proc sql; create table cp0 as select * from cp, metadata1(keep= sample_size noncase_fract); quit;

**%mend**;

**proc** **format**; * names for starting values;

value startvalues

**1**='standard'

**2**='high lambda'

**3**='low lambda'

**4**='high alpha'

**5**='low alpha'

**6**='high beta'

**7**='low beta'

; **run**;

**%macro** ***fitmod***;

* create datasets with missing values in case of non-convergence or other error causing non-

replacement of dataset;

data metadata2; minassayval_nat=**.**; maxassayval_nat=**.**; cases=**.**; sample_size=**.**; protection_curve='';

noncase_fract=**.**; starting_values=''; lambda_start=**.**; alpha_start=**.**; beta_start=**.**; beta_max=**.**;

nu_min=**.**; nu_max=**.**;

data parmests; Parameter=**.**; Estimate=**.**; StandardError=**.**;

data fitstats; Descr= ''; min2logL=**.**;

data convstat; Reason= ''; Status=**.**;

data hess;

data fitted0; assayval_nat=**.**; disease=**.**; logassayval=**.**; sample_size=**.**; noncase_fract=**.**; lambda=**.**;

Pred=**.**;

data gof1; disease=**.**; Pred=**.**; sum_yi=**.**; sum_i=**.**; y_bar_i=**.**;

data gof2; disease=**.**; sample_size=**.**; lambda=**.**; Pred=**.**; y_bar_i=**.**;

data gof3; sum_HosmLeme=**.**; GoF=**.**;

data t_p_data; t_50=**.**; t_80=**.**; t_90=**.**;

data ests; lambda= **.**; alpha= **.**; beta= **.**; nu= **.**; kappa=**.**; gamma=**.**;

data SEs; SE_lambda=**.**; SE_alpha= **.**; SE_beta=**.**; SE_nu=**.**; SE_kappa=**.**; SE_gamma=**.**;

data hessPD_0; COL1=**.**; data HessPD_1e_4; COL1=**.**;

data eigvals0; COL1=**.**; data eigvals1; eigen1=**.**; eigen2=**.**; eigen3=**.**; eigen4=**.**;

data HessPD; hessPD=**.**; hessPD_1e_4=**.**; eigen1=**.**; eigen2=**.**; eigen3=**.**; eigen4=**.**;

data res0; data res1; run;

* calculate starting values and bounds for parameters and develop model statements;

data metadata2; set metadata1;

startval=**1***&startvals; starting_values= put(startval,startvalues.);

if startval=**2** then lambda_start= min(**3.2***cases/sample_size,**.9**);

else if startval=**3** then lambda_start= **1.6***cases/sample_size;

else lambda_start= **2***cases/sample_size;

alpha_start= (log(maxassayval_nat)+log(minassayval_nat))/**2** + (startval=**4**) - (startval=**5**);

beta0=

( (index(protection_curve,'error')>**0**)***4.6527**

+ (index(protection_curve,'logistic')>**0**)***9.1902**

+ (index(protection_curve,'square_root_sigmoid')>**0**)***9.8494**

+ (index(protection_curve,'double_exponential')>**0**)***7.8240**

+ (index(protection_curve,'arctangent')>**0**)***63.641**

+ (index(protection_curve,'absolute_sigmoid')>**0**)***98**

+ (index(protection_curve,'generalized_symmetrical')>**0**)***98** )

/ (log(maxassayval_nat)-log(minassayval_nat));

beta_start= beta0 * (**2****(startval=**6**)) * (**0.5****(startval=**7**));

if index(protection_curve,'generalized_symmetrical')>**0** then beta_max= **1e12**; else beta_max= beta0***50**;

lam_al_be= compbl('lambda='||put(lambda_start,**8.6**)||' alpha='||put(alpha_start,**8.5**)||' beta='||

put(beta_start,**8.5**));

if index(protection_curve,'nonsymmetrical')>**0** then call

symput('ex','beta*(assayval_nat**nu*logassayval-alpha)');

else call symput('ex','beta*(logassayval-alpha)');

if index(protection_curve,'error')>**0** then call symput('pi_x',"cdf('normal',x)");

else if index(protection_curve,'logistic')>**0** then call symput('pi_x',"exp(x)/(1+exp(x))");

else if index(protection_curve,'square_root_sigmoid')>**0** then call symput('pi_x',"1/2*x/sqrt(1+x**2)+1/2");

else if index(protection_curve,'double_exponential')>**0** then call symput('pi_x',"(x>0)*(1-1/2*

exp(-x))+(x<0)*(1/2*exp(x))");

else if index(protection_curve,'arctangent')>**0** then call

symput('pi_x',"1/constant('pi')*atan(x)+1/2");

else if index(protection_curve,'absolute_sigmoid')>**0** then call

symput('pi_x',"1/2*x/(1+abs(x))+1/2");

if index(protection_curve,'generalized_symmetrical')>**0** then do;

call symput('parms',compbl(lam_al_be||' kappa=1'));

call symput('bounds',compbl('0 < beta < 1e12'));

call symput('pi_x',' 1/2*x/((1+abs(x)**kappa)**(1/kappa))+1/2'); end;

else if index(protection_curve,'incomplete')>**0** then do;

call symput('parms',compbl(lam_al_be||' gamma=0.8'));

call symput('bounds',compbl('0 < beta < '||put(beta_max,best8.)||', '||'0 < gamma <= 1')); end;

else if index(protection_curve,'nonsymmetrical')>**0** then do;

call symput('parms',compbl(lam_al_be||' nu=0'));

if maxassayval_nat>**1** then nu_min= -**1**/log(maxassayval_nat); else nu_min= -**1e6**;

if minassayval_nat<**1** then nu_max= -**1**/log(minassayval_nat); else nu_max= **1e6**;

call symput('bounds',compbl('0 < beta < '||put(beta_max,best8.)||', '||put(nu_min,best8.)||

' < nu < '||put(nu_max,best8.))); end;

else do;

call symput('parms',lam_al_be);

call symput('bounds',compbl('0 < beta < '||put(beta_max,best8.))); end;

if index(protection_curve,'incomplete')>**0** then call symput('p_prot','gamma*pi_x');

else call symput('p_prot','pi_x');

drop startval beta0 lam_al_be; run;

%put x= &ex;

%put pi_x= &pi_x;

%put p_prot= &p_prot;

%put parms= &parms;

%put bounds= &bounds;

* fit model;

ods listing close;

ods output ParameterEstimates=parmests ConvergenceStatus=convstat hessian=hess(drop= Row)

FitStatistics=fitstats(where= (Descr= '-2 Log Likelihood') rename= (Value= min2logL));

proc nlmixed data=cp0 gconv=**1e-12** hess corr;

parms &parms;

bounds &bounds;

x= &ex;

pi_x= &pi_x;

p_prot= &p_prot;

model disease ~ binary(**1**/(**1**+noncase_fract*(**1**/(lambda*(**1**-p_prot))-**1**)));

predict **1**/(**1**+noncase_fract*(**1**/(lambda*(**1**-p_prot))-**1**)) out=fitted0; id lambda;

run; quit; ods output close; ods listing;

* calculate goodness-of-fit, assay values for 50, 80 and 90% protection, and assemble results;

%***GoF***; %***t_p_dat***; %***assemble***;

**%mend**;

**%macro** ***GoF***; * calculate goodness-of-fit;

* calculate y_bar_i, the mean rate of disease for each assay value;

proc sort data=fitted0; by assayval_nat;

data gof1; set fitted0(keep= assayval_nat disease Pred); by assayval_nat; retain sum_yi sum_i **0**;

sum_yi= sum_yi + disease; sum_i= sum_i + **1**;

if last.assayval_nat then do; y_bar_i= sum_yi/sum_i; output; sum_yi=**0**; sum_i=**0**; end;

* merge back;

data gof2; merge fitted0(keep= assayval_nat disease Pred sample_size lambda)

gof1(keep= assayval_nat y_bar_i); by assayval_nat;

* calculate Hosmer Lemeshow statistic for each group and sum;

data gof3; set gof2; retain sum_yi sum_y_bar_i sum_Pred sum_i sum_HosmLeme **0** group **1**;

sum_yi = sum_yi + disease; sum_y_bar_i= sum_y_bar_i + y_bar_i;

sum_Pred= sum_Pred + Pred; sum_i= sum_i + **1**;

if _n_ ge group*sample_size/**10** then do;

if sum_Pred=**0** and abs(sum_y_bar_i-sum_Pred)<**1e-6** then HosmLeme=**0**;

else HosmLeme= ((sum_y_bar_i-sum_Pred)****2**)/sum_Pred/(**1**-sum_Pred/sum_i);

sum_HosmLeme= sum_HosmLeme + HosmLeme;

* calculate goodness-of-fit;

if _n_= sample_size then do; GoF= **1**-cdf('chisq',sum_HosmLeme,**10**-**3**); * using d.f. = G-3; output; end;

sum_yi=**0**; sum_y_bar_i=**0**; sum_Pred=**0**; sum_i=**0**; group=group+**1**; end; run;

**%mend**;

**%macro** ***t_p_dat***; * calculate assay values for 50, 80 and 90% protection;

data t_p_data; set fitted0(keep= logassayval Pred lambda noncase_fract) end=eof;

retain last_P_prot **0** last_logassayval -**1e6** t_50 t_80 t_90 **.** ;

P_prot= **1**-**1**/(lambda*((**1**/Pred-**1**)/noncase_fract+**1**));

if P_prot>**0.5** and last_P_prot<**0.5** then t_50= exp(last_logassayval+(**0.5**-last_P_prot)/

(P_prot-last_P_prot)*(logassayval-last_logassayval));

if P_prot>**0.8** and last_P_prot<**0.8** then t_80= exp(last_logassayval+(**0.8**-last_P_prot)/

(P_prot-last_P_prot)*(logassayval-last_logassayval));

if P_prot>**0.9** and last_P_prot<**0.9** then t_90= exp(last_logassayval+(**0.9**-last_P_prot)/

(P_prot-last_P_prot)*(logassayval-last_logassayval));

last_P_prot=P_Prot; last_logassayval=logassayval; if eof then output; keep t_50 t_80 t_90; run;

**%mend**;

**%macro** ***assemble***;

* format some results for assembly;

proc transpose data= parmests out= ests(drop= _NAME_); var Estimate; id Parameter; run;

proc transpose data= parmests prefix=se_ out=SEs(drop= _NAME_ _LABEL_); var StandardError; id

Parameter; run;

proc iml; use hess; read all into hess; eigvals= eigval(hess); posdef_1e_4= (all(eigvals>-**1e-4**));

create hessPD_1e_4 from posdef_1e_4; append from posdef_1e_4; quit;

proc iml; use hess; read all into hess; eigvals= eigval(hess); posdef= (all(eigvals>**0**));

create hessPD_0 from posdef; append from posdef; quit;

proc iml; use hess; read all into hess; eigvals= eigval(hess); create eigvals0 from eigvals;

append from eigvals; quit;

proc transpose data=eigvals0 out=eigvals1(drop= _name_) prefix= eigen; var col1; run;

data HessPD; merge hessPD_0(rename= (col1=hessPD)) hessPD_1e_4(rename= (col1=hessPD_1e_4)) eigvals1; run;

* assemble results;

data res0; merge convstat(rename= (Reason=convergence_status)) hessPD ests SEs fitstats

gof3(keep= sum_HosmLeme GoF) t_p_data;

* merge with metadata and calculate some assessment measures;

data res1; merge metadata2 res0;

conv_stat= **1**-Status; coef_var_lambda= SE_lambda/lambda; lambda_0_1= (**0**<lambda<**1**);

beta_0_99max= (**0**<beta<beta_max***0.99**); beta_rel= beta/beta_max;

if index(protection_curve,'nonsymmetrical')>**0** then nu_99minmax= (nu_min***0.99**<nu<nu_max***0.99**);

if index(protection_curve,'incomplete')>**0** then gamma_0_1=(**0.001**<gamma<**0.999**);

drop Descr Status; run;

* add to results file;

data results; set results res1; if not missing(protection_curve); format min2logL **8.3**; run;

**%mend**;

* Step 3: create null dataset to accumulate results;

**data** results; **run**;

* Step 4: select protection curve function - enter ONE of the following lines;

* Symmetrical two-parameter protection curves;

%let protection_curve= error;

%let protection_curve= logistic;

%let protection_curve= square_root_sigmoid;

%let protection_curve= double_exponential;

%let protection_curve= arctangent;

%let protection_curve= absolute_sigmoid;

* Generalized symmetrical protection curve;

%let protection_curve= generalized_symmetrical;

* ‘Incomplete protection’ protection curves;

%let protection_curve= incomplete_error;

%let protection_curve= incomplete_logistic;

%let protection_curve= incomplete_square_root_sigmoid;

%let protection_curve= incomplete_double_exponential;

%let protection_curve= incomplete_arctangent;

%let protection_curve= incomplete_absolute_sigmoid;

* Non-symmetrical protection curves

(note only the t_nu=t_N^nu*log(t_N) approach is implemented here);

%let protection_curve= nonsymmetrical_error;

%let protection_curve= nonsymmetrical_logistic;

%let protection_curve= nonsymmetrical_square_root_sigmoid;

%let protection_curve= nonsymmetrical_double_exponential;

%let protection_curve= nonsymmetrical_arctangent;

%let protection_curve= nonsymmetrical_absolute_sigmoid;

* Step 5: fit model;

%***fit_model***;

* Other protection curves may now be selected and the model fitted, results will accumulate in the

'results' file;

* Step 6: print results;

* metadata;

**proc** **print** data=results; var protection_curve starting_values minassayval_nat maxassayval_nat

sample_size cases noncase_fract; **run**;

* starting values and bounds on parameters;

**proc** **print** data=results; var protection_curve starting_values lambda_start alpha_start beta_start

beta_max nu_min nu_max; **run**;

* convergence and positive definite hessian;

**proc** **print** data=results; var conv_stat convergence_status hessPD hessPD_1e_4 eigen1 eigen2 eigen3

eigen4; **run**;

* or;

**proc** **print** data=results; var conv_stat convergence_status hessPD hessPD_1e_4 eigen1 eigen2 eigen3;

**run**;

* optimality criteria;

**proc** **print** data=results; var protection_curve starting_values min2logL sum_HosmLeme GoF

coef_var_lambda; **run**;

* parameters in the parameter space;

**proc** **print** data=results; var starting_values lambda lambda_0_1 beta beta_max beta_rel beta_0_99max

gamma gamma_0_1 nu nu_min nu_max nu_99minmax; **run**;

* parameters and SEs (based on observed information) - 1;

**proc** **print** data=results; var starting_values lambda SE_lambda alpha SE_alpha beta SE_beta beta_max

beta_rel beta_0_99max; **run**;

* parameters and SEs (SEs based on observed information) - 2 - as applicable;

**proc** **print** data=results; var starting_values kappa SE_kappa gamma SE_gamma gamma_0_1 nu SE_nu

nu_99minmax; **run**;

* assay values for 50, 80 and 90% protection;

**proc** **print** data=results; var protection_curve starting_values t_50 t_80 t_90; **run**;

* note: applications of the above code should be validated by independent parallel programming;
