# Supplementary material for: Some extensions in continuous models for immunological correlates of protection
Source: BMC Med Res Methodol. 2015 Dec 28;15:107. doi: 10.1186/s12874-015-0096-9 (PMC4692073; doi:10.1186/s12874-015-0096-9)
Supplement: Additional file 4: — Detail of results of fitting models with generalized symmetrical protection curve and with ‘incomplete protection’ protection curves. (DOCX 84 kb) [file 12874_2015_96_MOESM4_ESM.docx]

**Additional file 4: Detail of results of fitting** **models with generalized symmetrical protection curve and with ‘incomplete protection’ protection curves**

Parameter estimates and evaluation criteria for models with the generalized symmetrical protection curve and ‘incomplete protection’ protection curves fitted to the illustrative datasets are shown in the following table.

| Parameter estimate  −2×log-likelihood  Goodness-of-fit Coef. of Var. $\hat{\lambda}$ | Protection curve/function | | | | | | |
| --- | --- | --- | --- | --- | --- | --- | --- |
| Dataset (cases of disease:subjects) | Generalized sigmoid function | Incomplete protection curves | | | | | |
|  |  | Error function | Logistic function | Square root sigmoid | Double exponential | Arctangent function | Absolute sigmoid |
| German pertussis FHA IgG (44:1988) | $\hat{\kappa}$= 31.99 375.861 0.2740 0.1729 | - - - - | $\hat{\gamma}$= 0.906 372.505 0.0754 0.1600 | $\hat{\gamma}$= 0.907 372.264 0.0750 0.1599 | $\hat{\gamma}$= 0.906 372.414 0.0753 0.1600 | $\hat{\gamma}$= 0.908 372.060 0.0750 0.1598 | $\hat{\gamma}$= 0.908 372.042 0.0750 0.1598 |
| German pertussis PT IgG (44:1987) | $\hat{\kappa}$= 0.15 370.741 0.6903 0.1474 | $\hat{\gamma}$= 0.945 371.971 0.7631 0.1688 | $\hat{\gamma}$= 0.944 371.563 0.6905 0.1532 | $\hat{\gamma}$= 0.944 371.260 0.6947 0.1528 | $\hat{\gamma}$= 0.944 371.438 0.6944 0.1530 | $\hat{\gamma}$= 0.945 370.888 0.7030 0.1526 | $\hat{\gamma}$= 0.945 370.833 0.7056 0.1525 |
| German pertussis PRN IgG (44:1992) | $\hat{\kappa}$= 1.54 381.045 0.8270 0.1805 | - - - - | - - - - | - - - - | - - - - | - - - - | - - - - |
| German pertussis FIM IgG (44:1986) | $\hat{\kappa}$= 0.34 376.002 0.3379 0.1506 | - - - - | - - - - | - - - - | - - - - | - - - - | - - - - |
| German pertussis FHA IgA (44:1932) | $\hat{\kappa}$= 9.02 417.596 0.7178 0.1491 | - - - - | - - - - | - - - - | - - - - | - - - - | - - - - |
| German pertussis PT IgA (44:1933) | $\hat{\kappa}$= 16.69 418.058 0.4780 0.1490 | - - - - | - - - - | - - - - | - - - - | - - - - | - - - - |
| German pertussis PRN IgA (44:1968) | $\hat{\kappa}$= 8.71 407.263 0.2382 0.1491 | - - - - | - - - - | - - - - | - - - - | - - - - | - - - - |
| German pertussis FIM IgA (44:1994) | $\hat{\kappa}$= 0.12 402.667 0.0984 0.1496 | - - - - | - - - - | $\hat{\gamma}$= 0.818 403.483 0.1187 0.1587 | $\hat{\gamma}$= 0.814 403.929 0.1193 0.1592 | $\hat{\gamma}$= 0.824 402.700 0.1102 0.1579 | $\hat{\gamma}$= 0.824 402.629 0.1095 0.1579 |
| Piedra RSV/A (34:175) | $\hat{\kappa}$= 0.30 156.596 0.8661 0.1578 | $\hat{\gamma}$= 0.748 155.945 0.8968 0.2143 | $\hat{\gamma}$= 0.747 155.988 0.8946 0.2123 | $\hat{\gamma}$= 0.756 156.111 0.8885 0.2208 | $\hat{\gamma}$= 0.747 156.075 0.8910 0.2245 | $\hat{\gamma}$= 0.780 156.242 0.8831 0.2569 | $\hat{\gamma}$= 0.769 156.332 0.8803 0.2547 |
| Piedra RSV/B (34:175) | $\hat{\kappa}$= 1.92 154.413 0.6976 1.1648 | $\hat{\gamma}$= 0.885 154.047 0.7873 0.7112 | $\hat{\gamma}$= 0.897 154.154 0.7747 0.6783 | $\hat{\gamma}$= 0.940 154.289 0.7562 0.6702 | $\hat{\gamma}$= 0.905 154.333 0.7624 0.4608 | - - - - | - - - - |
| White/varicella (79:3459) | $\hat{\kappa}$= 2.35 641.582 1.0000 0.2284 | $\hat{\gamma}$= 0.973 641.628 1.0000 0.2367 | $\hat{\gamma}$= 0.980 641.578 1.0000 0.2276 | - - - - | $\hat{\gamma}$= 0.991 641.699 1.0000 0.2257 | - - - - | - - - - |
| Swedish pertussis FHA IgG (92:209) | $\hat{\kappa}$= 0.18 267.807 0.9574 0.1079 | $\hat{\gamma}$= 0.523 267.865 0.9490 0.1138 | $\hat{\gamma}$= 0.526 267.856 0.9503 0.1217 | $\hat{\gamma}$= 0.543 267.813 0.9544 0.1416 | $\hat{\gamma}$= 0.538 267.830 0.9538 0.1531 | $\hat{\gamma}$= 0.571 267.794 0.9564 0.1657 | $\hat{\gamma}$= 0.587 267.787 0.9574 0.2010 |
| Swedish pertussis PT IgG (92:209) | - - - - | $\hat{\gamma}$=0.616 267.207 0.9995 0.0779 | $\hat{\gamma}$= 0.616 267.207 0.9995 0.0779 | $\hat{\gamma}$= 0.616 267.209 0.9995 0.0779 | $\hat{\gamma}$= 0.616 267.207 0.9995 0.0779 | $\hat{\gamma}$= 0.617 267.209 0.9995 0.0779 | $\hat{\gamma}$= 0.617 267.209 0.9995 0.0779 |
| Swedish pertussis PRN IgG (92:209) | $\hat{\kappa}$= 0.12 241.176 0.8560 0.0816 | - - - - | $\hat{\gamma}$= 0.866 242.175 0.8814 0.0704 | $\hat{\gamma}$= 0.868 241.519 0.8762 0.0703 | $\hat{\gamma}$= 0.867 241.883 0.8796 0.0703 | $\hat{\gamma}$= 0.870 240.883 0.8698 0.0702 | $\hat{\gamma}$= 0.870 240.851 0.8695 0.0701 |
| Swedish pertussis FIM IgG (92:209) | $\hat{\kappa}$= 0.11 244.325 0.7639 0.0825 | $\hat{\gamma}$= 0.812 244.378 0.7774 0.0709 | $\hat{\gamma}$= 0.810 244.551 0.7781 0.0712 | $\hat{\gamma}$= 0.789 244.104 0.7871 0.0704 | $\hat{\gamma}$= 0.789 244.099 0.7872 0.0704 | $\hat{\gamma}$= 0.823 243.563 0.7038 0.0707 | $\hat{\gamma}$= 0.823 243.553 0.7032 0.0707 |
| Black Nicolay HAI (22:777) | $\hat{\kappa}$= 0.14 174.599 0.9180 0.2083 | $\hat{\gamma}$= 0.933 174.367 0.9323 0.2163 | $\hat{\gamma}$= 0.933 174.367 0.9323 0.2163 | $\hat{\gamma}$= 0.933 174.373 0.9322 0.2163 | $\hat{\gamma}$= 0.933 174.367 0.9323 0.2163 | $\hat{\gamma}$= 0.933 174.388 0.9318 0.2164 | $\hat{\gamma}$= 0.933 174.388 0.9317 0.2164 |

---

For some combinations of model and dataset the estimated ‘incomplete protection’ parameter$\hat{\gamma}$was greater than 1; MLEs were not considered to have been found in these instances.
