# Supplementary material for: Some extensions in continuous models for immunological correlates of protection
Source: BMC Med Res Methodol. 2015 Dec 28;15:107. doi: 10.1186/s12874-015-0096-9 (PMC4692073; doi:10.1186/s12874-015-0096-9)
Supplement: Additional file 5: — Detail of results of case-cohort investigation. (DOCX 41 kb) [file 12874_2015_96_MOESM5_ESM.docx]

**Additional file 5: Detail of results of case-cohort investigation**

The number of case-cohort datasets for which MLEs were found and the median standard error of the location parameter *α* based on the observed information in case-cohort datasets relative to its standard error when the model was fitted to the ‘all subjects’ illustrative dataset, are shown in the following table.

| Dataset  (cases of disease:subjects) | Number of case-cohort datasets for which MLEs found  (median SE_case-cohort_(α) ÷ SE_all-subjects_(α)) | | | | | |
| --- | --- | --- | --- | --- | --- | --- |
|  | Error function models | | | Absolute sigmoid models | | |
|  | Ratio ^*^ = 20 | Ratio ^*^ = 10 | Ratio ^*^ = 5 | Ratio ^*^ = 20 | Ratio ^*^ = 10 | Ratio ^*^ = 5 |
| German pertussis FHA IgG (44:1988) | 100 (1.035) | 100 (1.131) | 100 (1.287) | 100 (0.991) | 100 (1.040) | 100 (1.118) |
| German pertussis PT IgG (44:1987) | 97 (1.188) | 87 (1.287) | 65 (1.459) | 100 (1.211) | 100 (1.388) | 100 (1.423) |
| German pertussis PRN IgG (44:1992) | 100 (1.026) | 100 (1.023) | 100 (1.038) | 100 (1.018) | 100 (1.139) | 100 (1.064) |
| German pertussis FIM IgG (44:1986) | 100 (1.062) | 100 (1.264) | 99 (1.587) | 100 (0.875) | 100 (0.829) | 100 (0.771) |
| German pertussis FHA IgA (44:1932) | 50 (0.401) | 45 (0.400) | 49 (0.322) | 100 (2.742) | 100 (8.622) | 99 (14.10) |
| German pertussis PT IgA (44:1933) | 95 (0.985) | 90 (0.972) | 84 (0.900) | 100 (1.317) | 100 (1.994) | 100 (2.798) |
| German pertussis PRN IgA (44:1968) | - | - | - | 100 (1.043) | 100 (1.197) | 100 (2.293) |
| German pertussis FIM IgA (44:1994) | 99 (1.011) | 97 (1.108) | 92 (1.223) | 100 (1.008) | 100 (1.017) | 100 (1.084) |
| White/Varicella (79:3459) | 100 (1.049) | 100 (1.178) | 100 (1.311) | 100 (1.000) | 100 (1.030) | 100 (1.157) |
| Black Nicolay HAI (22:777) | 100 (1.013) | 100 (1.080) | 100 (1.213) | 100 (1.013) | 100 (1.045) | 100 (1.115) |
| ^*^ ratio of non-cases to cases | | | | | | |
