# Supplementary material for: Some extensions in continuous models for immunological correlates of protection
Source: BMC Med Res Methodol. 2015 Dec 28;15:107. doi: 10.1186/s12874-015-0096-9 (PMC4692073; doi:10.1186/s12874-015-0096-9)
Supplement: Additional file 6: — Detail of results for estimation of standard errors. (DOCX 59 kb) [file 12874_2015_96_MOESM6_ESM.docx]

**Additional file 6: Detail of results for estimation of standard errors**

*Bootstrap MLEs found*

MLEs with standard error estimates based on the observed information were found for 92,275 of the 108,000 combinations of bootstrap datasets and starting values. After selecting between starting values (which gave the same −2×log-likelihood within 10^−3^ 90.32% of the time) there were 46,591 unique bootstrap MLEs out of a possible 54,000.

*Estimates of standard error of* $\hat{\alpha}$

The number of bootstrap datasets for which MLEs were found, the median estimate of the standard error of $\hat{\alpha}$based on the observed information, the proportion of estimates between half and twice the median, and the standard deviation of the bootstrap estimates of $\hat{\alpha}$, are shown in the following tables, first for the non-parametric bootstraps and then for the parametric bootstraps.

| Illustrative dataset | Non-parametric bootstrap datasets | | | | | | | |
| --- | --- | --- | --- | --- | --- | --- | --- | --- |
|  | Error function models | | | | Absolute sigmoid models | | | |
|  | N* | Median $\mathrm{SE} \left(\hat{\alpha}\right)()$ | >½ and <2 × median | $\mathrm{SD} \left(\hat{\alpha}\right)()$ | N* | Median $\mathrm{SE} \left(\hat{\alpha}\right)()$ | >½ and <2 × median | $\mathrm{SD} \left(\hat{\alpha}\right)()$ |
| German pertussis FHA IgG | 949 | 0.932 | 0.712 | 0.992 | 1000 | 0.183 | 0.921 | 0.351 |
| German pertussis PT IgG | 668 | 2.572 | 0.510 | 1.717 | 1000 | 0.117 | 0.792 | 0.180 |
| German pertussis PRN IgG | 780 | 1.024 | 0.596 | 1.396 | 1000 | 0.117 | 0.702 | 0.627 |
| German pertussis FIM IgG | 775 | 1.276 | 0.724 | 1.150 | 1000 | 0.144 | 0.810 | 0.429 |
| German pertussis FHA IgA | 217 | 0.898 | 0.401 | 2.188 | 1000 | 0.124 | 0.478 | 0.686 |
| German pertussis PT IgA | 365 | 1.042 | 0.488 | 1.522 | 1000 | 0.109 | 0.527 | 0.571 |
| German pertussis PRN IgA | - | - | - | - | 1000 | 0.010 | 0.837 | 0.168 |
| German pertussis FIM IgA | 804 | 0.994 | 0.627 | 0.813 | 1000 | 0.061 | 0.610 | 0.314 |
| Piedra RSV/A | - | - | - | - | 929 | 0.486 | 0.801 | 0.625 |
| Piedra RSV/B | - | - | - | - | 833 | 0.549 | 0.838 | 0.984 |
| White/Varicella | 991 | 0.537 | 0.862 | 0.708 | 1000 | 0.098 | 0.798 | 0.260 |
| Swedish pertussis FHA IgG | - | - | - | - | - | - | - | - |
| Swedish pertussis PT IgG | 889 | 0.374 | 0.695 | 0.477 | 983 | 0.275 | 0.755 | 0.346 |
| Swedish pertussis PRN IgG | 975 | 0.329 | 0.984 | 0.312 | 1000 | 0.167 | 0.783 | 0.252 |
| Swedish pertussis FIM IgG | 748 | 0.852 | 0.656 | 0.646 | 991 | 0.329 | 0.864 | 0.342 |
| Black Nicolay HAI | 829 | 0.850 | 0.752 | 1.295 | 1000 | 0.344 | 0.834 | 0.301 |
| * number of bootstrap datasets for which MLEs found | | | | | | | | |

| Illustrative dataset | Parametric bootstrap datasets | | | | | | | |
| --- | --- | --- | --- | --- | --- | --- | --- | --- |
|  | Error function models | | | | Absolute sigmoid models | | | |
|  | N* | Median $\mathrm{SE} \left(\hat{\alpha}\right)()$ | >½ and <2 × median | $\mathrm{SD} \left(\hat{\alpha}\right)()$ | N* | Median $\mathrm{SE} \left(\hat{\alpha}\right)()$ | >½ and <2 × median | $\mathrm{SD} \left(\hat{\alpha}\right)()$ |
| German pertussis FHA IgG | 801 | 1.081 | 0.588 | 1.424 | 1000 | 0.191 | 0.840 | 0.416 |
| German pertussis PT IgG | 595 | 2.359 | 0.487 | 1.833 | 1000 | 0.122 | 0.793 | 0.294 |
| German pertussis PRN IgG | 826 | 1.008 | 0.570 | 1.515 | 1000 | 0.117 | 0.690 | 0.362 |
| German pertussis FIM IgG | 479 | 1.817 | 0.587 | 1.586 | 1000 | 0.152 | 0.847 | 0.329 |
| German pertussis FHA IgA | 194 | 1.372 | 0.448 | 2.174 | 1000 | 0.085 | 0.552 | 0.585 |
| German pertussis PT IgA | 232 | 1.457 | 0.496 | 1.545 | 1000 | 0.032 | 0.505 | 0.431 |
| German pertussis PRN IgA | - | - | - | - | 1000 | 0.005 | 0.640 | 0.113 |
| German pertussis FIM IgA | 656 | 0.913 | 0.584 | 1.014 | 999 | 0.068 | 0.682 | 0.185 |
| Piedra RSV/A | - | - | - | - | 964 | 0.509 | 0.831 | 0.862 |
| Piedra RSV/B | - | - | - | - | 877 | 0.587 | 0.857 | 0.917 |
| White/Varicella | 998 | 0.560 | 0.858 | 0.771 | 1000 | 0.103 | 0.826 | 0.163 |
| Swedish pertussis FHA IgG | - | - | - | - | - | - | - | - |
| Swedish pertussis PT IgG | 846 | 0.418 | 0.676 | 0.497 | 969 | 0.307 | 0.755 | 0.430 |
| Swedish pertussis PRN IgG | 951 | 0.352 | 0.964 | 0.394 | 1000 | 0.172 | 0.818 | 0.305 |
| Swedish pertussis FIM IgG | 697 | 0.822 | 0.660 | 0.749 | 991 | 0.368 | 0.859 | 0.561 |
| Black Nicolay HAI | 790 | 0.998 | 0.658 | 1.721 | 1000 | 0.314 | 0.611 | 0.556 |
| * number of bootstrap datasets for which MLEs found | | | | | | | | |

*Proportions of subjects below, above and within the 95% confidence interval for the assay value at which protection is 50%*

The proportions of subjects with assay values below, above and within the 95% confidence interval (CI) for the assay value at which protection is 50% are shown in the following tables, first for the error function models and then for the absolute sigmoid models. Confidence intervals are based on the mean of $\mathrm{SD} \left(\hat{\alpha}\right)()$ in parametric bootstraps and non-parametric bootstraps.

| Error function models  Dataset (cases of disease:subjects) | Estimated assay value at which protection is 50% (95% CI) | Proportions of subjects: | | |
| --- | --- | --- | --- | --- |
|  |  | below lower limit of CI (susceptible) | above upper limit of CI (protected) | within CI (undeter-mined) |
| German pertussis FHA IgG (44:1988) | 2.06 (0.19, 21.95) | 0.000 | 0.312 | 0.688 |
| German pertussis PT IgG (44:1987) | 0.16 (0.00, 5.12) | 0.000 | 0.429 | 0.571 |
| German pertussis PRN IgG (44:1992) | 4.89 (0.28, 84.68) | 0.000 | 0.169 | 0.831 |
| German pertussis FIM IgG (44:1986) | 0.42 (0.03, 6.20) | 0.000 | 0.291 | 0.709 |
| German pertussis FHA IgA (44:1932) | 8.92 (0.12, 641.3) | 0.001 | 0.000 | 0.999 |
| German pertussis PT IgA (44:1933) | 4.45 (0.22, 90.04) | 0.000 | 0.001 | 0.999 |
| German pertussis PRN IgA (44:1968) | - | - | - | - |
| German pertussis FIM IgA (44:1994) | 2.29 (0.38, 13.69) | 0.003 | 0.036 | 0.962 |
| Piedra RSV/A (34:175) | - | - | - | - |
| Piedra RSV/B (34:175) | - | - | - | - |
| White/Varicella (79:3459) | 1.58 (0.37, 6.75) | 0.033 | 0.722 | 0.246 |
| Swedish pertussis FHA IgG (92:209) | - | - | - | - |
| Swedish pertussis PT IgG (92:209) | 7.99 (3.08, 20.76) | 0.627 | 0.029 | 0.344 |
| Swedish pertussis PRN IgG (92:209) | 7.58 (3.80, 15.14) | 0.694 | 0.172 | 0.134 |
| Swedish pertussis FIM IgG (92:209) | 4.96 (1.26, 19.47) | 0.541 | 0.206 | 0.254 |
| Black Nicolay HAI (22:777) | 113.9 (5.93, 2189) | 0.190 | 0.118 | 0.691 |

| Absolute sigmoid models  Dataset (cases of disease:subjects) | Estimated assay value at which protection is 50% (95% CI) | Proportions of subjects: | | |
| --- | --- | --- | --- | --- |
|  |  | below lower limit of CI (susceptible) | above upper limit of CI (protected) | within CI (undeter-mined) |
| German pertussis FHA IgG (44:1988) | 2.10 (0.99, 4.46) | 0.251 | 0.578 | 0.171 |
| German pertussis PT IgG (44:1987) | 1.38 (0.87, 2.20) | 0.333 | 0.539 | 0.128 |
| German pertussis PRN IgG (44:1992) | 10.94 (4.15, 28.84) | 0.385 | 0.364 | 0.251 |
| German pertussis FIM IgG (44:1986) | 0.34 (0.16, 0.72) | 0.104 | 0.560 | 0.336 |
| German pertussis FHA IgA (44:1932) | 6.52 (1.88, 22.66) | 0.923 | 0.007 | 0.069 |
| German pertussis PT IgA (44:1933) | 4.36 (1.63, 11.63) | 0.692 | 0.003 | 0.305 |
| German pertussis PRN IgA (44:1968) | 2.52 (1.91, 3.32) | 0.696 | 0.080 | 0.225 |
| German pertussis FIM IgA (44:1994) | 3.52 (2.16, 5.74) | 0.244 | 0.139 | 0.617 |
| Piedra RSV/A (34:175) | 58.64 (13.66, 251.7) | 0.091 | 0.480 | 0.429 |
| Piedra RSV/B (34:175) | 58.91 (9.15, 379.4) | 0.051 | 0.611 | 0.337 |
| White/Varicella (79:3459) | 3.34 (2.20, 5.06) | 0.171 | 0.722 | 0.107 |
| Swedish pertussis FHA IgG (92:209) | - | - | - | - |
| Swedish pertussis PT IgG (92:209) | 5.50 (2.57, 11.77) | 0.536 | 0.091 | 0.373 |
| Swedish pertussis PRN IgG (92:209) | 6.03 (3.49, 10.42) | 0.689 | 0.206 | 0.105 |
| Swedish pertussis FIM IgG (92:209) | 5.10 (2.10, 12.35) | 0.656 | 0.249 | 0.096 |
| Black Nicolay HAI (22:777) | 107.6 (46.45, 249.0) | 0.358 | 0.516 | 0.126 |
